# Causes and attributable fraction of death from ARDS in inflammatory phenotypes of sepsis

**DOI:** 10.1186/s13054-024-04943-x

**Published:** 2024-05-14

**Authors:** Bruno Evrard, Pratik Sinha, Kevin Delucchi, Carolyn M. Hendrickson, Kirsten N. Kangelaris, Kathleen D. Liu, Andrew Willmore, Nelson Wu, Lucile Neyton, Emma Schmiege, Antonio Gomez, V. Eric Kerchberger, Ann Zalucky, Michael A. Matthay, Lorraine B. Ware, Carolyn S. Calfee

**Affiliations:** 1 Division of Pulmonary, Critical Care, Allergy and Sleep Medicine, Department of Medicine, University of California San Francisco, San Francisco, CA USA; 2 Inserm CIC 1435, Dupuytren Teaching Hospital, 87000 Limoges, France; 3 Division of Clinical and Translational Research, Washington University School of Medicine, Saint Louis, MO USA; 4 Department of Anesthesia, Division of Critical Care, Washington University, Saint Louis, MO USA; 5 Department of Psychiatry and Behavioral Sciences, University of California San Francisco, San Francisco, CA USA; 6 Division of Allergy, Pulmonary, and Critical Care Medicine, Department of Medicine, Zuckerberg San Francisco General Hospital and Trauma Center, San Francisco, CA USA; 7 Division of Hospital Medicine, Department of Medicine, University of California San Francisco, San Francisco, CA USA; 8 Division of Nephrology, Department of Medicine, University of California San Francisco, San Francisco, CA USA; 9 Department of Anesthesia, University of California San Francisco, San Francisco, CA USA; 10 Cardiovascular Research Institute, University of California San Francisco, San Francisco, CA USA; 11 Division of Allergy, Pulmonary, and Critical Care Medicine, Department of Medicine, Vanderbilt University Medical Center, Nashville, TN USA; 12 Department of Biomedical Informatics, Vanderbilt University Medical Center, Nashville, TN USA; 13 Department of Pathology, Microbiology and Immunology, Vanderbilt University Medical Center, Nashville, TN USA

**Keywords:** Respiratory distress syndrome, Acute lung injury, Sepsis, Phenotype, Mortality, Cause of death

## Abstract

**Background:**

Hypoinflammatory and hyperinflammatory phenotypes have been identified in both Acute Respiratory Distress Syndrome (ARDS) and sepsis. Attributable mortality of ARDS in each phenotype of sepsis is yet to be determined. We aimed to estimate the population attributable fraction of death from ARDS (PAF_ARDS_) in hypoinflammatory and hyperinflammatory sepsis, and to determine the primary cause of death within each phenotype.

**Methods:**

We studied 1737 patients with sepsis from two prospective cohorts. Patients were previously assigned to the hyperinflammatory or hypoinflammatory phenotype using latent class analysis. The PAF_ARDS_ in patients with sepsis was estimated separately in the hypo and hyperinflammatory phenotypes. Organ dysfunction, severe comorbidities, and withdrawal of life support were abstracted from the medical record in a subset of patients from the EARLI cohort who died (n = 130/179). Primary cause of death was defined as the organ system that most directly contributed to death or withdrawal of life support.

**Results:**

The PAF_ARDS_ was 19% (95%CI 10,28%) in hypoinflammatory sepsis and, 14% (95%CI 6,20%) in hyperinflammatory sepsis. Cause of death differed between the two phenotypes (p < 0.001). Respiratory failure was the most common cause of death in hypoinflammatory sepsis, whereas circulatory shock was the most common cause in hyperinflammatory sepsis. Death with severe underlying comorbidities was more frequent in hypoinflammatory sepsis (81% vs. 67%, p = 0.004).

**Conclusions:**

The PAF_ARDS_ is modest in both phenotypes whereas primary cause of death among patients with sepsis differed substantially by phenotype. This study identifies challenges in powering future clinical trials to detect changes in mortality outcomes among patients with sepsis and ARDS.

**Supplementary Information:**

The online version contains supplementary material available at 10.1186/s13054-024-04943-x.

## Background

Acute respiratory distress syndrome (ARDS) is characterized by mortality of 35–45% [1] and considerable heterogeneity, contributing to the current challenge of developing effective treatment [2]. Two molecular phenotypes of ARDS, hypo- and hyperinflammatory, have been identified based largely on plasma levels of biomarkers reflecting inflammation, epithelial and endothelial injury and coagulation abnormalities [2–4]. Specifically, the hyperinflammatory phenotype, which represents about one-third of ARDS cases, is associated with high levels of inflammatory biomarkers, increased use of vasopressors, and higher mortality rates [2–4]. In contrast, the hypoinflammatory phenotype, representing approximately two-thirds of ARDS cases, is associated with lower levels of inflammatory biomarkers and reduced mortality rates [2–4]. These phenotypes have also been identified in sepsis, with similar characteristics, prognosis and differential response to activated protein C, suggesting this schema captures phenotypes of critical illness overall and not only ARDS [5, 6].

The attributable fraction and population attributable fraction are epidemiological tools useful for estimating the potential impact of an exposure on an outcome. Population attributable fraction describes the reduction in the rate of the outcome if the exposure could be completely removed, assuming the exposure is causal. These metrics have been used in other fields to inform feasibility and design of trials [7]; for example, if population AF_ARDS_ is low, sample size requirements for clinical trials in ARDS with primary outcome of mortality will be quite high [8]. The attributable fraction of death from sepsis-associated ARDS (AF_ARDS_) is the proportion of deaths attributable to ARDS among all deaths in patients who developed sepsis-associated ARDS. The population AF_ARDS_ (PAF_ARDS_) in this context is the proportion of deaths that would be prevented following elimination of ARDS in patients with sepsis [9]. In a previous study, Auriemma et al. reported the PAF_ARDS_ to be 16% and 18% in two independent cohorts of septic adults, with mortality mainly driven by severe ARDS (P/F ratio < 100) [10]. However, this study encompassed both hypo- and hyperinflammatory phenotypes and used sepsis patients without ARDS as the reference population. More recently, Saha et al. estimated the attributable mortality of ARDS phenotypes using a completely different reference population (either critically ill patients without acute respiratory failure or patients with a unilateral radiographic infiltrate), and without estimating the PAF_ARDS_ [11]. The PAF_ARDS_ for hyper- and hypoinflammatory phenotypes using a referent population of sepsis patients remains unknown. Moreover, whether causes of death differ in sepsis based on molecular phenotypes is also unknown and may inform the proportions of mortality that may be modifiable in each phenotype. In this study, we aimed to (1) estimate 60 days in hospital PAF_ARDS_ in patients with hypo-inflammatory versus hyperinflammatory sepsis, and (2) to determine causes of death in hypo-inflammatory versus hyper-inflammatory sepsis to further contextualize our PAF_ARDS_ analyses.

## Methods

### Participants

We studied patients from two prospectively enrolled cohorts of critically ill adults: (1) the Early Assessment of Renal and Lung Injury (EARLI) study, which enrolls adults admitted from the emergency department to the intensive care unit (ICU) at either an academic medical center or safety net hospital in San Francisco, California, and (2) the Validating Acute Lung Injury markers for Diagnosis (VALID) study which enrolls critically ill adults from an academic medical center in Nashville, Tennessee.

### Sepsis and ARDS definition

We selected patients admitted to the ICU for sepsis [12]. Because data collection started prior to publication of the Sepsis 3 definition [13], sepsis was defined as documented or suspected infection in the presence of two or more characteristics of the systemic inflammatory response syndrome within the first two days of ICU admission [12]. Presence or absence of sepsis, and pulmonary or non-pulmonary origin of sepsis if present, was meticulously assessed by a participating study physician using all the data available from the patient’s hospitalization. Patients were defined as having ARDS if they met Berlin criteria for ARDS on at least one of the first five hospital days for EARLI and between ICU days one through four in VALID [14]. Day one was defined as the admission date in the emergency department in EARLI, whereas it was defined as the day of ICU admission in VALID. Development of ARDS was adjudicated by at least two study physicians and by review of all chest radiographs during the first 5 days of enrollment, using criteria set forth by the AECC or Berlin criteria [14, 15]. When patients met chest radiograph and oxygenation criteria for ARDS, then the medical record was thoroughly reviewed for any evidence of a primary or contributory cardiogenic cause of pulmonary edema. We additionally identified patients who were not receiving mechanical ventilation but who met the American-European Consensus Conference (AECC) criteria for acute lung injury (ALI) during the same time frame [15]. Further information on exclusions criteria is provided in Additional file 1: E-methods. Latent class assignments for included patients were determined in a previous study [6]. Briefly, latent class analysis (LCA) is a statistical technique that uses mixture modelling to find the best fitting model for a set of data, based on the hypothesis that the data contain several unobserved groups or classes [16].

EARLI was approved by the University of California, San Francisco Institutional Review Board (IRB) and VALID by the Vanderbilt IRB. Consent was obtained from patients or their surrogates when possible, as previously described [10].

### Determination of the cause of the death

We determined cause of death in patients who died in the EARLI cohort for whom electronic health records (EHR) were available. Patients’ data were reviewed by one trained intensivist who did not participate in ARDS adjudication and was blinded to ARDS and phenotype status. Rigorous inspection of the temporal relationship of laboratory data, imaging data, hemodynamic, respiratory parameters and physician’s notes, using a standardized case ascertainment template (Additional file 1: E-methods) [17], was carried out to define cause of death. If determination of cause was challenging, adjudication was done with a second trained intensivist (CSC). 25% of randomly patients were assessed by a third intensivist (AZ) to determine inter-rater reliability.

For each patient, we reviewed the medical record for evidence of dysfunction of eight organ systems during the 72 h prior to death (Additional file 1: Table E1). We classified organ dysfunction as severe or irreversible using modified definitions from prior studies (Additional file 1: Table E1) [17, 18]. The primary cause of death was defined as the organ system that most directly contributed to death or withdrawal of life support (Additional file 1: Figure E1). Further information regarding determination of cause of death is provided in the E-methods.

**Table 1:** Characteristics of combined cohort of EARLI and VALID patients on admission stratified by Phenotype and presence of ARDS

	Hypoinflammatory n = 1168			Hyperinflammatory n = 569		
	ARDS n = 440*	No ARDS n = 728*	p-value†	ARDS n = 272*	No ARDS n = 297*	p-value†
Age (years)	60 (48, 73)	61 (50, 71)	0.7	59 (51, 70)	61 (51, 69)	> 0.9
Male	251 (57%)	422 (58%)	0.7	148 (54%)	156 (53%)	0.7
SAPS II	50 (36, 64)	43 (32, 56)	< 0.001	66 (52, 82)	54 (42, 69)	< 0.001
Modified APACHE II	24 (19, 30)	22 (17, 27)	< 0.001	30 (24, 37)	27 (22, 33)	< 0.001
Diabetes	127 (29%)	237 (33%)	0.2	64 (24%)	78 (26%)	0.5
Congestive heart failure	91 (21%)	114 (16%)	0.027	46 (17%)	44 (15%)	0.5
Coronary artery disease	51 (12%)	114 (16%)	0.053	25 (9%)	43 (14%)	0.052
Stroke	43 (10%)	75 (10%)	0.8	15 (6%)	17 (6%)	> 0.9
Chronic liver disease	17 (4%)	48 (7%)	0.049	60 (22%)	61 (21%)	0.7
Chronic kidney disease	83 (19%)	148 (20%)	0.5	31 (11%)	73 (25%)	< 0.001
Pulmonary sepsis	343 (78%)	321 (44%)	< 0.001	150 (55%)	75 (25%)	< 0.001
Vasopressors	190 (43%)	267 (37%)	0.027	209 (77%)	218 (73%)	0.3
Fluid administration in Emergency Department (L)	2.6 (1.3, 4.6)	3.0 (1.6, 5.0)	0.010	3.5 (2.0, 5.4)	3.5 (2.0, 6.2)	0.2
Albumin (g/L)	2.7 (2.2, 3.1)	2.7 (2.3, 3.1)	0.4	2.3 (1.8, 2.7)	2.4 (2.0, 2.8)	0.028
Hematocrit (%)	31 (27, 35)	30 (26, 35)	0.7	26 (23, 31)	26 (23, 31)	0.6
Creatinine (mg/L)	1.28 (0.87, 2.23)	1.34 (0.88, 2.35)	0.5	1.72 (1.06, 2.64)	2.34 (1.34, 4.00)	< 0.001
Bicarbonate (mmol/L)	23.0 (20.0, 26.0)	23.0 (20.0, 26.0)	0.6	18.0 (15.0, 21.0)	19.0 (16.0, 22.0)	0.009
Protein C (%)	67 (40, 99)	71 (50, 98)	0.034	39 (25, 65)	36 (22, 55)	0.025
IL-6 (pg/mL)	49 (18, 135)	29 (12, 99)	< 0.001	919 (185, 4,968)	239 (65, 1,070)	< 0.001
IL-8 (pg/mL)	12 (6, 25)	13 (7, 27)	0.2	213 (61, 1,260)	97 (26, 344)	< 0.001
Invasive Mechanical ventilation	289 (66%)	340 (47%)	< 0.001	195 (72%)	126 (42%)	< 0.001
PaO2/FiO2 (mmHg)	127 (75, 194)	209 (134, 330)	< 0.001	126 (75, 183)	233 (139, 353)	< 0.001
SpO2/FiO2	148 (96, 213)	239 (158, 329)	< 0.001	153 (96, 223)	240 (155, 332)	< 0.001
ARDS severity‡						
Mild	105 (24%)	–	–	58 (21%)	–	–
Moderate	172 (39%)	–	–	109(40%)	–	–
Severe	163 (37%)	–	–	105 (39%)	–	–
ICU stay of length (days) *§*	6 (4, 10)	4 (2, 6)	< 0.001	8 (5, 16)	5 (3, 8)	< 0.001
Ventilation length (days) *§*	3 (0, 7)	0 (0, 3)	< 0.001	5 (0, 10)	0 (0, 3)	< 0.001
In hospital mortality to 60 days	108 (25%)	95 (13%)	< 0.001	143 (53%)	101 (34%)	< 0.001

*Median (IQR); n (%)

^†^Wilcoxon rank sum test; Pearson's Chi-squared test

^‡^At the time of ARDS onset

^§^For survivors only

**Fig. 1:**
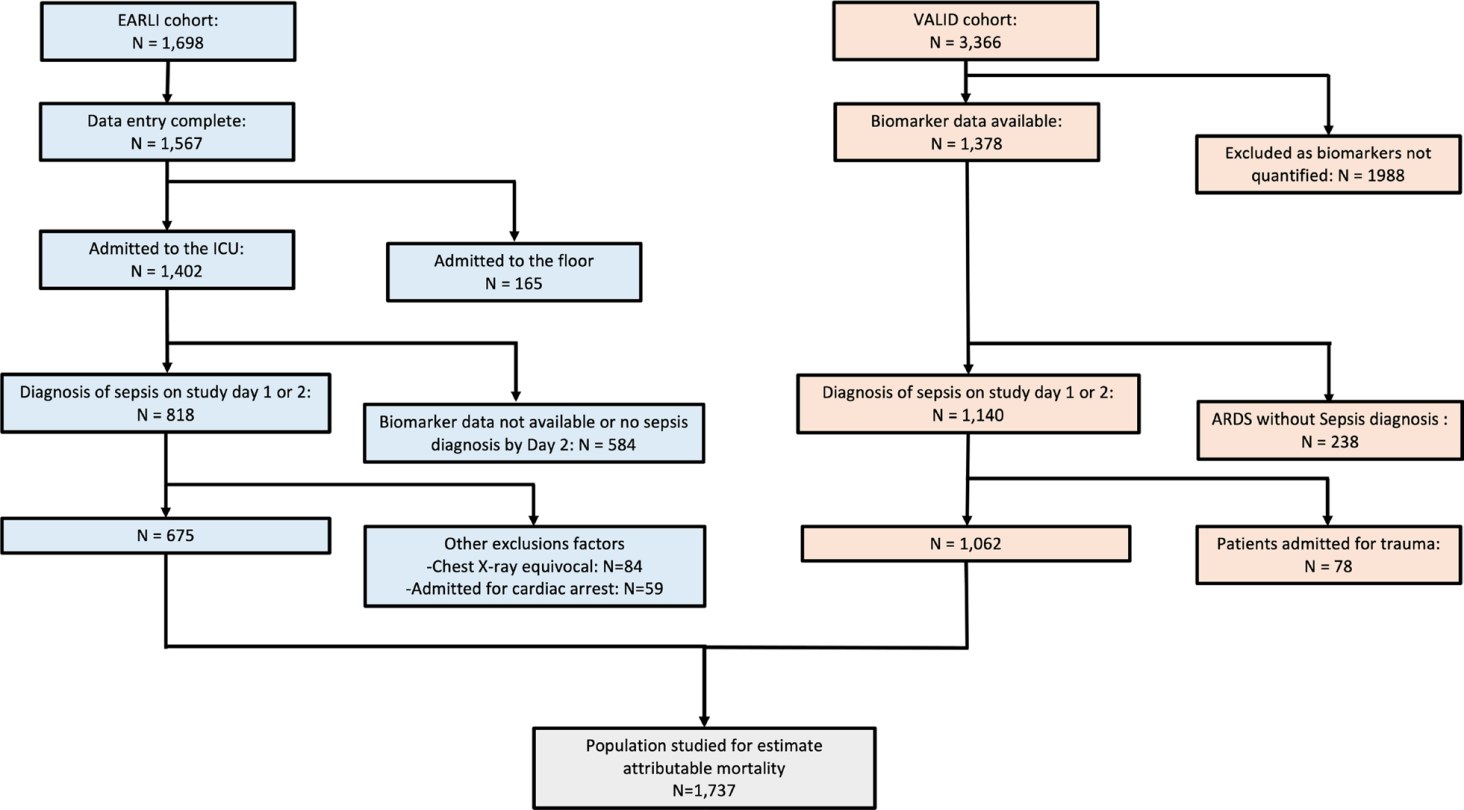
Flow chart of the study

### Statistical analysis

Sample size estimation is provided in the E-methods (Additional file 1: E-methods) and was used to support the decision to combine EARLI and VALID for most analyses. Pearson’s chi square and Wilcoxon rank sum test were used to compare baseline variables stratified by phenotypes of sepsis. The primary outcome was in-hospital 60-day mortality. AF_ARDS_ and PAF_ARDS_ were estimated within each phenotype separately; specifically, the mortality of hypoinflammatory sepsis with ARDS was compared to the mortality of hypoinflammatory sepsis without ARDS, without considering hyperinflammatory patients, and vice versa. To estimate the AF_ARDS_ and the PAF_ARDS_ within each phenotype of sepsis, we used methods outlined previously [9, 10, 19]. Estimates were based on indirect standardization, which computes the weighted average of stratum-specific estimates in the reference population, using weights from the study population [10, 19]. Strata were defined by modified APACHE II quartiles; the oxygenation component of APACHE II was removed for this analysis. We also conducted multiple sensitivity analyses, one of which involved a matching approach using propensity scores, for which we used a directed acyclic graph to determine the variables to include in the model (Additional file 1: Figure E2). Details of the sensitivity analysis are provided in the E-methods (Additional file 1: E-methods). Pearson’s Chi Square was used to compare cause of death between patients with hypoinflammatory vs hyperinflammatory sepsis and with or without ARDS. A p-value less than 0.05 was considered statistically significant. Analyses were performed using the STDRATE procedure in SAS (Version 3.81) for the calculation of AF_ARDS_ and the PAF_ARDS_ using strata method and using R (Version 4.2.2) for all other analysis.

**Fig. 2:**
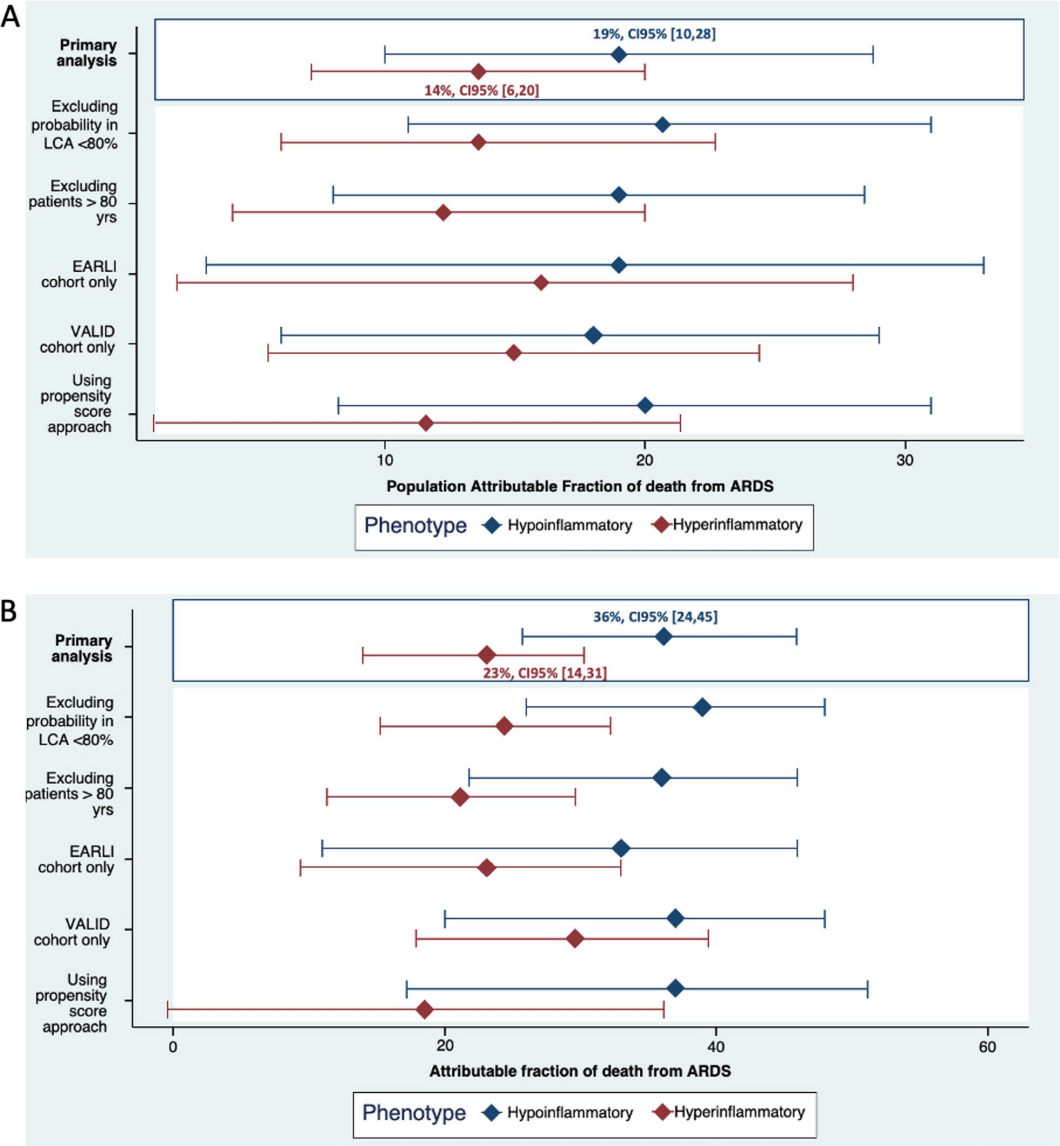
**A** Estimation with 95% confidence interval and sensitivity analysis of the population attributable fraction of death from ARDS in each phenotype of sepsis. **B** Estimation with 95% confidence interval and sensitivity analysis of the attributable fraction of death from ARDS in each phenotype of sepsis. *ARDS* Acute respiratory distress syndrome,* LCA* Latent class analysis

## Results

### Patient characteristics

Overall, 1737 patients were included, 675 from EARLI and 1062 from VALID (Fig. 1). Patients from EARLI were significantly older (Median: 66 years, IQR [55,78] vs. 58 years, IQR [47,67], p < 0.001), more frequently required vasopressors (58% vs. 47%, p < 0.001), and less frequently required invasive mechanical ventilation (45% vs. 61%, p < 0.001) (Additional file 1: Table E2) compared to patients from VALID. The proportion of patients who developed ARDS within five days of enrollment was also higher in EARLI (47% vs. 37%, p < 0.001). In-hospital overall mortality was similar in both cohorts (27% vs. 25%, p = 0.5) and also comparable within the ARDS subgroup in both cohorts (37% vs. 34%, p = 0.5). In both cohorts, more than 85% patients who developed ARDS did so on day 1 or day 2 of study enrollment (Additional file 1: Figure E3A).

**Table 2:** Characteristics of EARLI patients collected from the day of the death or of withdrawal of care, and cause of the death stratified by phenotype

	Overall sepsis (N = 130)			ARDS subgroup (N = 85)		
	Hypoinflammatory, N = 54*	Hyperinflammatory, N = 76*	p-value†	Hypoinflammatory, N = 30*	Hyperinflammatory, N = 55*	p-value†
Full code on admission	32 (59%)	59 (79%)	0.053	17 (57%)	44 (81%)	0.016
Withdrawal of life support	38 (76%)	50 (70%)	0.5	22 (79%)	39 (75%)	0.7
Modified SOFA score‡	5 (3, 9)	12 (9, 15)	< 0.001	6 (4, 8)	13 (11, 15.0)	< 0.001
Multiorgan failure n(%)	34 (71%)	64 (86%)	0.034	18 (75%)	47 (89%)	0.2
Main organ failure involved in the death			< 0.001			< 0.001
Circulatory failure	15 (28%)	48 (63%)		6 (20%)	37 (67%)	
Respiratory failure	32 (59%)	11 (14%)		20 (67%)	9 (16%)	
Other	7 (13%)	17 (22%)		4 (13%)	9 (16%)	
Main severe comorbidities			0.004			0.042
No underlying severe comorbidities	*10 (19%)*	*25 (33%)*		*8 (27%)*	*22 (40%)*	
Elderly	15 (28%)	12 (16%)		8 (27%)	10 (18%)	
Severe lung disease	10 (19%)	2 (3%)		6 (20%)	1 (2%)	
Advanced malignancy	14 (26%)	23 (30%)		5 (17%)	13 (24%)	
Other severe	*5 (9%)*	*14 (18%)*		*3 (10%)*	*9 (16%)*	
Time to death (days)	8 (5, 14)	6 (2, 13)	0.10	7 (4, 12)	6 (2, 13)	0.3

*Median (IQR); n (%)

^†^Wilcoxon rank sum test; Pearson's Chi-squared test

^‡^Score without neurological component

**Fig. 3:**
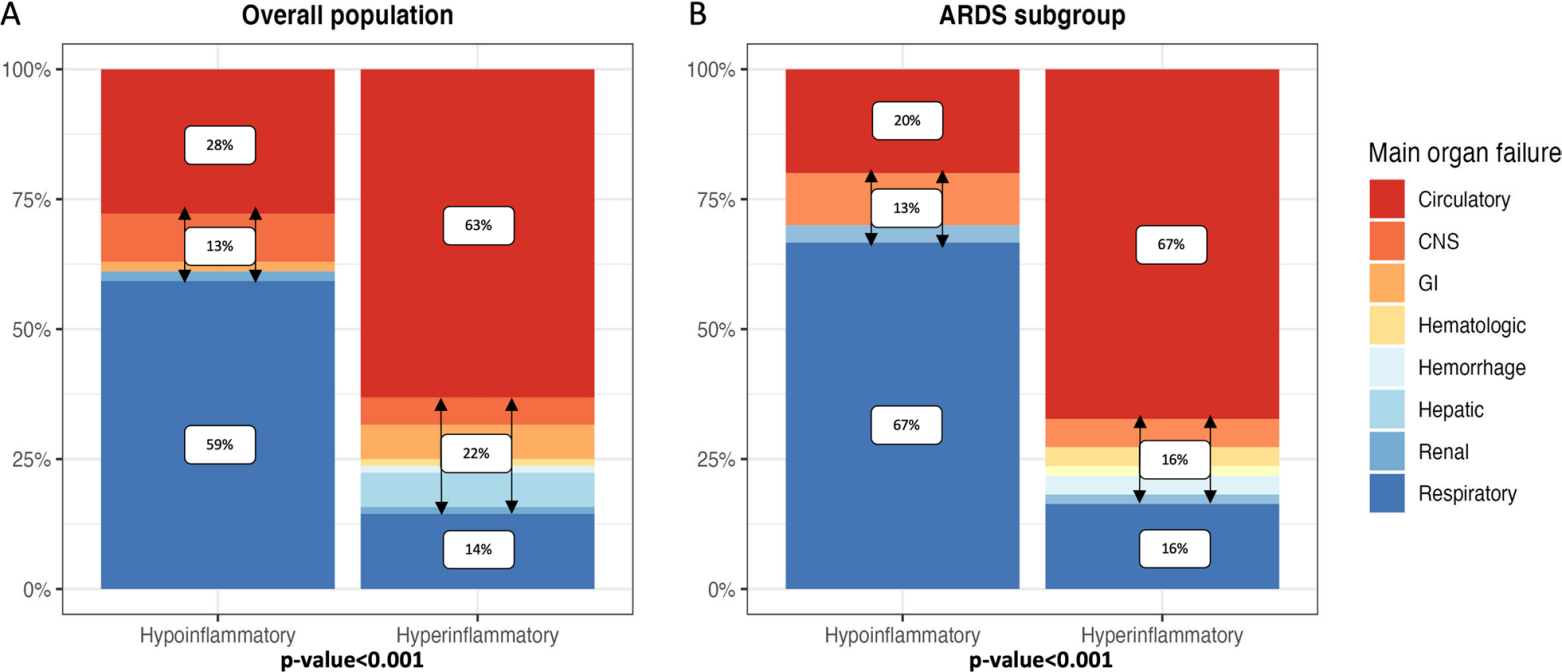
**A** Barplot showing the comparison of the cause of death between hypoinflammatory and hyperinflammatory sepsis in EARLI overall population. **B** Barplot showing the comparison of the cause of death between hypoinflammatory and hyperinflammatory sepsis in the subgroup of patients who developed ARDS. *ARDS* Acute respiratory distress syndrome,* CNS* Central nervous system,* GI* Gastro-intestinal

Considering both cohorts together, 1168 patients (67%) were allocated to the hypoinflammatory group, and 440 of these (37%) developed ARDS during their study observation period (Table 1, Additional file 1: Figure E4). Age and sex were similar between patients developing ARDS and those who did not, whereas proportion requiring vasopressors, pulmonary sepsis, modified APACHE II and in-hospital mortality were higher in those who developed ARDS (Table 1). Albumin levels and hematocrit were similar in patients who developed ARDS and those who did not, whereas patients without ARDS received more fluids in the emergency department (Table 1). Among hypoinflammatory patients who died, 41 (20%) died before the end of the ARDS ascertainment time frame (5 days) without having developed ARDS (Additional file 1: Figure E3B).

In the combined cohorts, 569 patients (33%) were allocated to the hyperinflammatory phenotype, and 272 of these (48%) developed ARDS in the five days following their ICU admission (Table 1 and Additional file 1: Figure E4). As in the hypoinflammatory phenotype, proportion of pulmonary sepsis, modified APACHE II and in-hospital mortality were higher in patients who developed ARDS. The proportion of patients requiring vasopressors was similar between those who developed ARDS and those who did not. Albumin levels were slightly lower in patients who developed ARDS, while the volume of fluids received in the emergency department and hematocrit did not differ (Table 1). Among hyperinflammatory patients who died, 55 (23%) died before the end of the ARDS ascertainment time frame (5 days) without having developed ARDS (Additional file 1: Figure E3B).

### AFARDS and population AFARDS

In hypoinflammatory sepsis, the AF_ARDS_ was 36% (95%CI: 24,45%), and the PAF_ARDS_ was 19% (95%CI: 10,28%) (Fig. 2). This finding indicates that eliminating ARDS in hypoinflammatory sepsis would provide a relative mortality reduction of 19%. Sensitivity analyses excluding older patients, those with intermediate probability of phenotype membership, and using propensity scores did not meaningfully alter the results (Additional file 1: Tables E3 and E4).

In hyperinflammatory sepsis, the AF_ARDS_ was 23% (95%CI: 14,31%) and the PAF_ARDS_ was 14% (95% CI: 1,23%), indicating that eliminating ARDS would provide a relative mortality reduction of 14% in hyperinflammatory sepsis (Fig. 2). Similar to hypoinflammatory sepsis, sensitivity analyses did not meaningfully alter the results (Additional file 1: Tables E3 and E4).

### Cause of death

Among the 179 patients who died in EARLI, 49 (27%) were excluded from analysis because no electronic medical record data was available to determine cause of death (mainly patients enrolled from 2008 to 2011). Of the 130 studied, 54 were hypoinflammatory and 76 were hyperinflammatory. Inter-rater reliability for cause of death was excellent (Kappa = 0.94, p < 0.001).

Cause of death differed by phenotype (p < 0.001) (Additional file 1: Figs. 3A-B and E5; Table 2). In hypoinflammatory sepsis, patients died mainly from respiratory failure (59%) (Fig. 3A), which was primarily characterized by failure to wean from respiratory support rather than refractory hypoxemia (Additional file 1: Figure E6). In contrast, patients who died in the hyperinflammatory group died mainly from circulatory failure (63%) (Fig. 3A and Additional file 1: Figure E7). When considering only patients who developed ARDS, these proportions and differences remained similar (Fig. 3B). Among patients who died, 53% of patients with hypoinflammatory sepsis died in the ICU versus 73% in the hyperinflammatory phenotype (p = 0.018). Underlying severe comorbidities were present in most patients but were more pronounced in hypoinflammatory sepsis: 33% of hyperinflammatory sepsis patients who died had no underlying severe comorbidities, versus 19% in hypoinflammatory sepsis (p = 0.004) (Table 2; Additional file 1: Figure E5). In the overall sepsis population and in the ARDS subgroup, modified SOFA score collected on day of death or day of withdrawal of life support was significantly lower in patients with hypoinflammatory sepsis compared to patients with hyperinflammatory sepsis (Table 2 and Additional file 1: Table E5). Further details comparing cause of death of patients who developed ARDS and those who did not in each phenotype are provided in the Additional file 1.

## Discussion

To our knowledge, this study estimates for the first time the AF_ARDS_ and PAF_ARDS_ in inflammatory phenotypes of sepsis. While the PAF_ARDS_ was relatively similar in hyper- and hypo-inflammatory sepsis, cause of death differed substantially between the phenotypes. Death in hypoinflammatory sepsis was mainly driven by respiratory causes, most commonly failure to wean from respiratory support, and death in hyperinflammatory sepsis was mainly driven by circulatory failure/shock.

Our analyses of cause of death in each phenotype identified several patterns of interest. First, we found that patients in the hyperinflammatory phenotype died mainly because of circulatory failure (refractory shock). We could not determine if circulatory failure was caused directly by effects of sepsis on the peripheral vasculature (e.g. vasoplegia, hypovolemia) or by pulmonary vascular dysfunction leading to right ventricular failure, which is frequently present in patients who die from ARDS [20–22], or a combination of the two. Second, we found that patients with hypoinflammatory sepsis died mainly because of respiratory failure, regardless of the presence of ARDS. Respiratory failure in these cases was not driven by irreversible hypoxemia but by failure to wean from ventilatory or oxygenation support. Third, more than 80% of patients with hypoinflammatory sepsis who died had severe comorbidities which contributed to the decision to withdraw or not escalate life support. Thus, deaths in hypoinflammatory sepsis may reflect at least in part a population with severe comorbidities that limit functional recovery from critical illness. Numerous studies have reported that patients with ARDS frequently die because of extrapulmonary organ failure [17, 18, 23], but to our knowledge, the finding that patients with hypoinflammatory sepsis died mainly because of failure to wean from respiratory support is novel.

The PAF_ARDS_ can be defined as the proportion of death over a specified time that would be prevented following elimination of the exposure (i.e., ARDS) in the sepsis population, assuming the exposure is causal [9]. Following this definition, 19% of deaths could be avoided during hospitalization if ARDS were eliminated in hypoinflammatory sepsis, and 14% of deaths in hyperinflammatory sepsis. Surprisingly, the AF_ARDS_ and PAF_ARDS_ seemed relatively similar and perhaps even lower in hyperinflammatory sepsis compared to hypoinflammatory sepsis. One possible explanation for this finding is that in hyperinflammatory sepsis, the lung is only one of many failing organs; thus, treating respiratory failure is less likely to eliminate risk of death. Another explanation could be that more patients with hyperinflammatory sepsis die before they can develop ARDS. However, as ARDS occurred mainly in the first 48 h, and because only a small proportion of patients died without ARDS within the five first days, this explanation seems less likely. As a result of this low PAF_ARDS_, therapies that target lung-specific pathways in hyperinflammatory sepsis may require dramatic efficacy to identify a mortality benefit, while therapies that have less organ-specific effects may be more fruitful [8, 10].

The AF_ARDS_ and PAF_ARDS_ in hypoinflammatory sepsis were modestly higher, with a lower prevalence of multi-system organ failure, which might imply that ARDS plays a larger role in short-term mortality in this phenotype. However, the proportion of hypoinflammatory patients who died with a high burden of severe comorbidities was very high. If confirmed by other studies, these findings may limit the utility of mortality as an endpoint for future studies in hypoinflammatory sepsis, especially when severe comorbidities persist and contribute to a short life expectancy. Using severe comorbidities as a surrogate for frailty, we speculate that the modifiable proportion of death in this phenotype may be lower. It is important to emphasize that we did not explore other important endpoints such as morbidity, quality of life and other patient-centered outcomes, or the financial impact of ARDS in patients who survived [24]. Taken together, these findings highlight challenges to achieving mortality reduction in ARDS clinical trials. Designs for future trials in both phenotypes should take account of these findings, which could indicate that a large number of patients would need to be treated in order to identify a survival benefit, or that the trial population must be more strictly selected [7, 10, 23]. Cooperative multinational trials may be required in order to generate studies adequately powered for mortality endpoints.

With recent data suggesting that hypoinflammatory and hyperinflammatory phenotypes are generalizable to sepsis [5, 6], we chose to study the AF_ARDS_ and PAF_ARDS_ within each sepsis phenotype. This approach considers ARDS as a complication of each phenotype of sepsis, rather than considering each phenotype of ARDS as a complication of overall sepsis or more broadly of critical illness. Analyses that assess the PAF of hyperinflammatory ARDS and hypoinflammatory ARDS relative to an unselected control group (i.e., unselected patients, or ventilated controls) will likely find quite different results. In a previous study, Saha et al. estimated the attributable fraction of mortality from hyperinflammatory ARDS using a different control population (either critically ill patients without acute respiratory failure or patients with a unilateral radiographic infiltrate) [11]. In contrast with our results, they found that the AF of death from hyperinflammatory ARDS was higher than from hypoinflammatory ARDS. The observed difference may be explained by the presence of both inflammatory phenotypes within their control population.

This study has several strengths. First unlike some prior studies [11], phenotypes were assigned by LCA, a robust method [16] with consistent and well-replicated findings [2, 5, 25–27]. Moreover, sensitivity analysis provided similar results, even when using another approach (propensity scoring) to estimate attributable mortality. Second, we strictly followed established methodological guidelines for estimation and interpretation of PAF_ARDS_ [28]. Third, it included two large, diverse prospective cohorts from distinct centers which provide a generalizable population with external validity to estimate PAF_ARDS_. Fourth, all patients were meticulously assessed for both sepsis and ARDS. Fifth, inter-rater reliability for cause of death was excellent.

This study also has limitations. First, we only explored cause of death in EARLI, and some patients had missing data due to timing of EHR implementation. Second, we used the Sepsis-2 criteria to define sepsis, since studies started before the Sepsis-3 definition. However, as we enrolled only critically ill patients, it is unlikely that our patients would not fulfill the more recent criteria for sepsis [13]. Third, we were not able to assess if LCA class assignment changed over time, although a previous analysis showed that ARDS phenotypes were stable over the first 3 days [29]. Fourth, while the high burden of comorbidities in hypoinflammatory patients may imply a higher prevalence of frailty, we do not have formal measures of frailty, which might shed further light on causes of death in patients with multi-comorbidity [30]. Fifth, we assumed that no ARDS patients were misclassified for the analysis. However, our systematic prospective approach to determine presence of ARDS by at least two specialists limits the risk of classification bias, and we explicitly excluded patients whose ARDS diagnosis was unclear or equivocal in one cohort. Sixth, we did not treat ARDS as a time-varying exposure, which could theoretically lead to an overestimation of the population AF_ARDS_ [31, 32]. However, this potential bias is limited by the fact that the vast majority of ARDS occurred on Day 1 or 2 of study enrollment. Finally, we focused here only on sepsis patients admitted in ICU, and findings may not be generalizable to patients with other risk factors for ARDS.

## Conclusion

This study provides important new findings about PAF_ARDS_ in each inflammatory phenotype of sepsis. The PAF_ARDS_ was modest (< 20%) in both phenotypes and relatively similar. Patients with ARDS in hypoinflammatory sepsis died primarily from respiratory failure with a high burden of severe comorbidities contributing to decisions around end-of-life. Conversely, patients with ARDS in hyperinflammatory sepsis died primarily from circulatory failure. These findings suggest that identifying effective therapies to reduce mortality from sepsis-induced ARDS may be challenging in both phenotypes but for different reasons—namely, the higher prevalence of multiorgan failure in hyperinflammatory sepsis which may decrease the impact of treating only one organ, and the burden of comorbidities which may impact short-term prognosis for patients with hypoinflammatory sepsis.

### Supplementary Information

**Additional file 1. E-methods:** Participants, exclusion criteria, determination of the cause of the death and statistical analysis. **E-results**. **Table E1:** Definition of severe and irreversible organ system dysfunction derived from Stapleton et al. and Ketcham et al. **Table E2:** Characteristics on ICU admission between EARLI and VALID cohort. **Table E3:** Estimation of population attributable fraction of death from ARDS in each subphenotype of sepsis. **Table E4:** Estimation of attributable fraction of death from ARDS in each subphenotype of sepsis. **Table E5:** Details of the SOFA score without neurologic component before the day of death or at the time of withdrawal of life support, stratified by phenotype. **Table E6:** Characteristics of patients before the day of death or at the time of the withdrawal of life support, stratified by subphenotype and presence or not of ARDS. **Figure E1:** Algorithm from Ketcham et al. to determinate the primary cause of death. **Figure E2:** Directed Acyclic graph used for propensity score matching. **Figure E3:** Barplot showing the day of diagnosis of ARDS from ICU admission (Day 1) in each subphenotype of sepsis, and showing the proportion of patient who died in each phenotype and stratified by the timing of death. **Figure E4:** Overview of the study. **Figure E5:** Alluvial plot showing the relation between severe comorbidities, the origin of sepsis the phenotype of sepsis, the presence or not of ARDS and the cause of death, stratified by the subphenotype of sepsis. **Figure E6:** Upset plot showing the number of patients with one or multiple irreversible or severe organ dysfunction collected at the time of death or the withdrawal of life support in hypoinflammatory sepsis using the standardized case ascertainment template. **Figure E7:** Upset plot showing the number of patients with one or multiple irreversible or severe organ dysfunction collected at the time of death or the withdrawal of life support in hyperinflammatory sepsis using the standardized case ascertainment template. **Figure E8:** Results for the matching using propensity score in the hypoinflammatory sepsis. **Figure E9:** Results for the matching using propensity score in the hyperinflammatory sepsis. **E-references**. **STROBE Statement**.

## Data Availability

The datasets used and/or analyzed during the current study are available from the corresponding author on reasonable request.

## Abbreviations

ARDS: Acute respiratory distress syndrome
AF_ARDS_: Attributable fraction of death from ARDS
PAF_ARDS_: Population attributable fraction of death from ARDS
ICU: Intensive care unit
APACHE II: Acute Physiology and Chronic Health Evaluation II
EARLI: Early Assessment of Renal and Lung Injury cohort
VALID: Validating Acute Lung Injury markers for Diagnosis cohort

## References

[1] Bellani G, Laffey JG, Pham T, Fan E, Brochard L, Esteban A (2016). Epidemiology, patterns of care, and mortality for patients with acute respiratory distress syndrome in intensive care units in 50 countries. JAMA.

[2] Calfee CS, Delucchi K, Parsons PE, Thompson BT, Ware LB, Matthay MA (2014). Subphenotypes in acute respiratory distress syndrome: latent class analysis of data from two randomised controlled trials. Lancet Respir Med.

[3] Bos LD, Schouten LR, van Vught LA, Wiewel MA, Ong DSY, Cremer O (2017). Identification and validation of distinct biological phenotypes in patients with acute respiratory distress syndrome by cluster analysis. Thorax.

[4] Sinha P, Churpek MM, Calfee CS (2020). Machine learning classifier models can identify acute respiratory distress syndrome phenotypes using readily available clinical data. Am J Respir Crit Care Med.

[5] Sinha P, He J, Delucchi K, Zhuo H, Abbott J, Jones C, et al. Latent class analysis-derived hypoinflammatory and hyperinflammatory phenotypes are generalisable to sepsis patients requiring intensive care. In: B95 ARDS WHATS LATEST Gt. American Thoracic Society; 2022. p. A3431–A3431.

[6] Sinha P, Kerchberger VE, Willmore A, Chambers J, Zhuo H, Abbott J (2023). Identifying molecular phenotypes in sepsis: an analysis of two prospective observational cohorts and secondary analysis of two randomised controlled trials. Lancet Respir Med..

[7] Shankar-Hari M, Harrison DA, Rowan KM, Rubenfeld GD (2018). Estimating attributable fraction of mortality from sepsis to inform clinical trials. J Crit Care.

[8] Muscedere JG, Day A, Heyland DK (2010). Mortality, attributable mortality, and clinical events as end points for clinical trials of ventilator-associated pneumonia and hospital-acquired pneumonia. Clin Infect Dis.

[9] Rockhill B, Newman B, Weinberg C (1998). Use and misuse of population attributable fractions. Am J Public Health.

[10] Auriemma CL, Zhuo H, Delucchi K, Deiss T, Liu T, Jauregui A (2020). Acute respiratory distress syndrome-attributable mortality in critically ill patients with sepsis. Intensive Care Med.

[11] Saha R, Pham T, Sinha P, Maddali MV, Bellani G, Fan E (2023). Estimating the attributable fraction of mortality from acute respiratory distress syndrome to inform enrichment in future randomised clinical trials. Thorax.

[12] Levy MM, Fink MP, Marshall JC, Abraham E, Angus D, Cook D (2003). 2001 SCCM/ESICM/ACCP/ATS/SIS international sepsis definitions conference. Intensive Care Med.

[13] Singer M, Deutschman CS, Seymour CW, Shankar-Hari M, Annane D, Bauer M (2016). The third international consensus definitions for sepsis and septic shock (sepsis-3). JAMA.

[14] Ranieri VM, Rubenfeld GD, Thompson BT, Ferguson ND, Caldwell E, ARDS Definition Task Force (2012). Acute respiratory distress syndrome: the Berlin definition. JAMA.

[15] Bernard GR, Artigas A, Brigham KL, Carlet J, Falke K, Hudson L (1994). Definitions, mechanisms, relevant outcomes, and clinical trial coordination. Am J Respir Crit Care Med.

[16] Sinha P, Calfee CS, Delucchi KL (2021). Practitioner’s guide to latent class analysis: methodological considerations and common pitfalls. Crit Care Med.

[17] Ketcham SW, Sedhai YR, Miller HC, Bolig TC, Ludwig A, Co I (2020). Causes and characteristics of death in patients with acute hypoxemic respiratory failure and acute respiratory distress syndrome: a retrospective cohort study. Crit Care.

[18] Stapleton RD, Wang BM, Hudson LD, Rubenfeld GD, Caldwell ES, Steinberg KP (2005). Causes and timing of death in patients with ARDS. Chest.

[19] Wacholder S, Benichou J, Heineman EF, Hartge P, Hoover RN (1994). Attributable risk: advantages of a broad definition of exposure. Am J Epidemiol.

[20] Vieillard-Baron A, Naeije R, Haddad F, Bogaard HJ, Bull TM, Fletcher N (2018). Diagnostic workup, etiologies and management of acute right ventricle failure. Intensive Care Med.

[21] Evrard B, Goudelin M, Giraudeau B, François B, Vignon P. Right ventricular failure is strongly associated with mortality in patients with moderate-to-severe COVID-19-related ARDS and appears related to respiratory worsening. Intensive Care Med. 2022. 10.1007/s00134-022-06730-0 PMC9098148 35552780

[22] Vieillard-Baron A, Cecconi M (2014). Understanding cardiac failure in sepsis. Intensive Care Med.

[23] Villar J, Martínez D, Mosteiro F, Ambrós A, Añón JM, Ferrando C (2018). Is overall mortality the right composite endpoint in clinical trials of acute respiratory distress syndrome?. Crit Care Med.

[24] Herridge MS, Moss M, Hough CL, Hopkins RO, Rice TW, Bienvenu OJ (2016). Recovery and outcomes after the acute respiratory distress syndrome (ARDS) in patients and their family caregivers. Intensive Care Med.

[25] Sinha P, Delucchi KL, Chen Y, Zhuo H, Abbott J, Wang C (2022). Latent class analysis-derived subphenotypes are generalisable to observational cohorts of acute respiratory distress syndrome: a prospective study. Thorax.

[26] Calfee CS, Delucchi KL, Sinha P, Matthay MA, Hackett J, Shankar-Hari M (2018). Acute respiratory distress syndrome subphenotypes and differential response to simvastatin: secondary analysis of a randomised controlled trial. Lancet Respir Med.

[27] Famous KR, Delucchi K, Ware LB, Kangelaris KN, Liu KD, Thompson BT (2017). Acute respiratory distress syndrome subphenotypes respond differently to randomized fluid management strategy. Am J Respir Crit Care Med.

[28] Pérez-Ríos M, Rey-Brandariz J, Galán I, Fernández E, Montes A, Santiago-Pérez MI (2022). Methodological guidelines for the estimation of attributable mortality using a prevalence-based method: the STREAMS-P tool. J Clin Epidemiol.

[29] Delucchi K, Famous KR, Ware LB, Parsons PE, Thompson BT, Calfee CS (2018). Stability of ARDS subphenotypes over time in two randomised controlled trials. Thorax.

[30] Fronczek J, Polok K, de Lange DW, Jung C, Beil M, Rhodes A (2021). Relationship between the Clinical Frailty Scale and short-term mortality in patients ≥ 80 years old acutely admitted to the ICU: a prospective cohort study. Crit Care.

[31] Von Cube M, Schumacher M, Putter H, Timsit J, Van De Velde C, Wolkewitz M (2020). The population-attributable fraction for time-dependent exposures using dynamic prediction and landmarking. Biom J.

[32] Steen J, Vansteelandt S, DeBus L, Depuydt P, Gadeyne B, Benoit DD (2021). Attributable mortality of ventilator-associated pneumonia. Replicating findings, revisiting methods. Ann Am Thorac Soc.

