# Supplementary material for: Causes and attributable fraction of death from ARDS in inflammatory phenotypes of sepsis
Source: Crit Care. 2024 May 14;28:164. doi: 10.1186/s13054-024-04943-x (PMC11092165; doi:10.1186/s13054-024-04943-x)
Supplement: Supplementary file 1 — Additional file 1. E-methods: Participants, exclusion criteria, determination of the cause of the death and statistical analysis. E-results. Table E1: Definition of severe and irreversible organ system dysfunction derived from Stapleton et al. and Ketcham et al. Table E2: Characteristics on ICU admission between EARLI and VALID cohort. Table E3: Estimation of population attributable fraction of death from ARDS in each subphenotype of sepsis. Table E4: Estimation of attributable fraction of death from ARDS in each subphenotype of sepsis. Table E5: Details of the SOFA score without neurologic component before the day of death or at the time of withdrawal of life support, stratified by phenotype. Table E6: Characteristics of patients before the day of death or at the time of the withdrawal of life support, stratified by subphenotype and presence or not of ARDS. Figure E1: Algorithm from Ketcham et al. to determinate the primary cause of death. Figure E2: Directed Acyclic graph used for propensity score matching. Figure E3: Barplot showing the day of diagnosis of ARDS from ICU admission (Day 1) in each subphenotype of sepsis, and showing the proportion of patient who died in each phenotype and stratified by the timing of death. Figure E4: Overview of the study. Figure E5: Alluvial plot showing the relation between severe comorbidities, the origin of sepsis the phenotype of sepsis, the presence or not of ARDS and the cause of death, stratified by the subphenotype of sepsis. Figure E6: Upset plot showing the number of patients with one or multiple irreversible or severe organ dysfunction collected at the time of death or the withdrawal of life support in hypoinflammatory sepsis using the standardized case ascertainment template. Figure E7: Upset plot showing the number of patients with one or multiple irreversible or severe organ dysfunction collected at the time of death or the withdrawal of life support in hyperinflammatory sepsis using the standardized case ascertainme [file 13054_2024_4943_MOESM1_ESM.docx]

Causes and attributable fraction of death from ARDS in inflammatory subphenotypes of sepsis

Online Data Supplement

Bruno Evrard, Pratik Sinha, Kevin Delucchi, Carolyn M. Hendrickson, Kirsten N. Kangelaris, Kathleen D. Liu, Andrew Willmore, Nelson Wu, Emma Schmiege, Antonio Gomez, Eric Kerchberger, Ann Zalucky, Michael A. Matthay, Lorraine B. Ware, Carolyn S. Calfee

**Authors Affiliations:**

1 Division of Pulmonary, Critical Care, Allergy and Sleep Medicine, Department of Medicine, University of California San Francisco, San Francisco, CA, USA

2 Inserm CIC 1435, Dupuytren Teaching Hospital, Limoges 87000-F, France

3 Division of Clinical and Translational Research, Washington University School of Medicine, Saint Louis, MO, USA

4 Department of Anesthesia, Division of Critical Care, Washington University, Saint Louis, MO, USA

5 Department of Psychiatry and Behavioral Sciences, University of California San Francisco, San Francisco, CA, USA

6 Division of Allergy, Pulmonary, and Critical Care Medicine, Department of Medicine, Zuckerberg San Francisco General Hospital and Trauma Center, San Francisco, CA, USA

7 Division of Hospital Medicine, Department of Medicine, University of California San Francisco, San Francisco, CA, USA

8 Division of Nephrology, Department of Medicine, University of California San Francisco, San Francisco, CA USA

9 Department of Anesthesia, University of California San Francisco, San Francisco, CA, USA

10 Cardiovascular Research Institute, University of California San Francisco, San Francisco, CA, USA

11 Division of Allergy, Pulmonary, and Critical Care Medicine, Department of Medicine, Vanderbilt University Medical Center, Nashville, TN, USA

12 Department of Biomedical Informatics, Vanderbilt University Medical Center, Nashville, TN, USA

13 Department of Pathology, Microbiology and Immunology, Vanderbilt University Medical Center, Nashville, TN, USA

Corresponding author: [bruno.evrard@chu-limoges.fr](mailto:bruno.evrard@chu-limoges.fr)

Table of contents

[E-methods 5](#_Toc165382756)

[Participants 5](#_Toc165382757)

[Exclusion criteria 5](#_Toc165382758)

[Determination of the cause of the death 5](#_Toc165382759)

[Statistical analysis 7](#_Toc165382760)

[*Sample size* 8](#_Toc165382761)

[*Sensitivity analysis* 8](#_Toc165382762)

[*Population attributable fraction and attributable fraction using propensity score* 8](#_Toc165382763)

[*Inter-rater reliability* 9](#_Toc165382764)

[E-results 10](#_Toc165382765)

[E-Tables 11](#_Toc165382766)

[Table E1: Definition of severe and irreversible organ system dysfunction derived from Stapleton et al. and Ketcham et al. 11](#_Toc165382767)

[Table E2: Characteristics on ICU admission between EARLI and VALID cohort 12](#_Toc165382768)

[Table E3: Estimation of population attributable fraction of death from ARDS in each subphenotype of sepsis 14](#_Toc165382769)

[Table E4: Estimation of attributable fraction of death from ARDS in each subphenotype of sepsis 15](#_Toc165382770)

[Table E5: Details of the SOFA score without neurologic component before the day of death or at the time of withdrawal of life support, stratified by phenotype 16](#_Toc165382771)

[Table E6: Characteristics of patients before the day of death or at the time of the withdrawal of life support, stratified by subphenotype and presence or not of ARDS 17](#_Toc165382772)

[E-Figures 18](#_Toc165382773)

[Figure E1: 18](#_Toc165382774)

[Figure E2: 19](#_Toc165382775)

[Figure E3 20](#_Toc165382776)

[Figure E4: 21](#_Toc165382777)

[Figure E5 22](#_Toc165382778)

[Figure E6 23](#_Toc165382779)

[Figure E7 24](#_Toc165382780)

[Figure E8: 25](#_Toc165382781)

[Figure E9: 26](#_Toc165382782)

[E-references 27](#_Toc165382783)

[STROBE Statement 28](#_Toc165382784)

# E-methods

## Participants

Code status at admission was assessed in EARLI based on documented preferences in medical records at the time of ICU admission. We used APACHE II and SAPS II to assess the severity of illness.

Latent class assignments for included patients were determined in a previous study [1]. Briefly, a combination of continuous and categorical variables were used for the LCA modelling [2]. Average probabilities for class assignment were generated for each observation. Patients were assigned to a phenotype if the LCA-generated probability for that phenotype was 0.5 or greater. Further information for the LCA determination is provided in the original manuscript [1].

## Exclusion criteria

We excluded from the analysis patients admitted for out of hospital cardiac arrest (because of their high mortality and difficulties ascertaining ARDS), patients admitted for trauma, patients with chest radiographs deemed equivocal for ARDS diagnosis [3], and patients directly admitted to the hospital floor.

## Determination of the cause of the death

#### Organ system dysfunction

Since all included patients had sepsis, we removed from our assessment the concept of “sepsis” as an organ failure [4, 5]. We preferentially defined patients with shock (of any etiology) as patients suffering from circulatory failure. Moreover, because of the absence of specific hemodynamic assessment collected for the study allowing to better assess the cause of the circulatory failure, we also integrated the “cardiac dysfunction” feature inside the class of circulatory failure. We also modified the definition of severe neurologic dysfunction as patients with a persistent coma defined by a Glasgow coma scale < 8 after 3 days without any sedation. If a patient underwent withdrawal of life support before meeting any of the objective organ dysfunction criteria, we assigned irreversible dysfunction to the organ system primarily responsible for the decision to withdraw life support to accurately capture cause of death. We defined multi-organ failure as severe organ dysfunction in at least two organ systems.

#### Cause, features, and circumstances of death

For each patient, we assessed (i) the primary organ system responsible for death, (ii) whether withdrawal of life support occurred prior to death, (iii) the time between the onset of sepsis or ARDS and death, (iv) SOFA score without the neurological component on the day of death or when the decision for withdrawal of life support was made, and (v) if the death or the decision of withdrawal of life support was related to the patient’s severe comorbidities. As in previous studies, the primary cause of death was defined as the organ dysfunction that most directly resulted in the patient’s death or the decision to withdraw care [4, 5].

Withdrawal of life support and rationale for this decision was determined from clinical documentation of intent to withdraw life support and/or not escalate life support in the event of clinical decompensation and subsequent removal or non-escalation of life-sustaining interventions. Severe comorbidities were defined as end-stage malignancies or chronic disease (e.g., lung fibrosis, advanced COPD, Amyotrophic Lateral Sclerosis, advanced cirrhosis), age > 80, and/or the accumulation of several severe chronic conditions reported in the medical record.

#### Standardized case ascertainment template

|  |  | **Possible Value** |
| --- | --- | --- |
|  | id |  |
| Code status | Code on admission | Full/DNI/DNR |
|  | Code on hospitalisation | Full/DNI/DNR |
|  | date_code | Month/Day/Year |
| Details of SOFA | cv_sofa | 0-4 |
|  | res_sofa | 0-4 |
|  | coag_sofa | 0-4 |
|  | liver_sofa | 0-4 |
|  | renal_sofa | 0-4 |
| Irreverisible organ dysfunction | irr_circulatory | 0-1 |
|  | irr_pulm | 0-1 |
|  | irr_neuro | 0-1 |
|  | irr_hemato | 0-1 |
|  | irr_hemorr | 0-1 |
|  | irr_hepatic | 0-1 |
|  | irr_gi | 0-1 |
|  | irr_renal | 0-1 |
| Severe organ dysfunction | sev_circulatory | 0-1 |
|  | sev_pulm | 0-1 |
|  | sev_neuro | 0-1 |
|  | sev_hemato | 0-1 |
|  | sev_hemorr | 0-1 |
|  | sev_hepatic | 0-1 |
|  | sev_gi | 0-1 |
|  | sev_renal | 0-1 |
| Severe comorbidities | severe_comorbidity1 | Free text |
|  | severe_comorbidity2 | Free text |
|  | severe_comorbidity3 | Free text |
|  | final_cause | Circulatory/pulm/neuro/hemato/hemorr  Hepatic/gi/renal |
|  | final_severecomorbidity | Free text |

## **Statistical analysis**

### *Sample size*

We first estimated the sample size needed in each subphenotype of sepsis for the estimation of the population AF_ARDS_ [6]. Given the absence of prior studies assessing the ARDS proportion within different sepsis phenotypes and the associated mortality rates within each subgroup, we posited the following estimations. For an estimation of population AF_ARDS_ of 15% in hypoinflammatory sepsis, based on the incidence of death of 17%, a prevalence of ARDS of 33% and an alpha at 5% and a power at 80%, at least 1306 patients with hypoinflammatory sepsis were required in this group. Likewise, for an estimation of population AF_ARDS_ of 15% in hyperinflammatory sepsis, based on the incidence of death of 43%, a prevalence of ARDS of 45% and an alpha at 5% and a power at 80%, at least 507 patients were required in this group.

### *Sensitivity analysis*

We conducted several sensitivity analyses: (1) estimating population AF_ARDS_ in VALID and EARLI cohort separately, (2) excluding patients over 80 years old, (3) excluding patients with a probability of LCA class below 80%, and (4) using a propensity score based on APACHE II and age in lieu of the indirect standardization method [7].

### *Population attributable fraction and attributable fraction using propensity score*

Propensity score was calculated for each patient to estimate that patient’s probability to develop ARDS. We used a directed acyclic graph (Figure E2) to select the variables to include in the model. We included for propensity-score calculation a modified APACHE II score without oxygenation parameter, the origin of sepsis (pulmonary versus non-pulmonary), presence of diabetes, cirrhosis, or shock on ICU admission. Due to the low proportion of missing data (only 4 for the modified APACHE II score), the data were assumed to be missing at random and analyses were used for all models. Then, using the “MatchIt” package, a nearest neighbor algorithm without replacement and with a caliper width of 0.2 was used for propensity-score matching using logistic regression with a 1:1 ratio in the hyperinflammatory subphenotype and a 1:2 ratio in the hypoinflammatory phenotype: each patient with ARDS was matched with 1 patient or 2 patients (respectively in hyper-and hypoinflammatory subphenotype) without ARDS with the nearest propensity-score. We assessed balance post matching by estimating standardized mean differences and examining empirical cumulative density function plots for the variables used for matching. This matching specification yielded good balance, as indicated in Figures E8 and E9. 927/1168 patients were matched in the hypoinflammatory subphenotypes, whereas 406/569 were matched in the hyperinflammatory subphenotype. We then estimated the population attributable fraction of death from ARDS using the “epiR” package in each subphenotype.

### *Inter-rater reliability*

Inter-rater reliability was assessed by Cohen’s kappa.

# E-results

*Cause of death*

Among the 54 patients who died in the hypoinflammatory group, 30 (56%) developed ARDS. The proportion of patients admitted with Full code status was similar among those who developed ARDS and those who did not (57% versus 62%, p>0.9) (Table E5), and the SOFA score without neurological component was similar (6, IQR:4,8 versus 4, IQR: 2,8, p=0.2). Time to death was not significantly different in patients who developed ARDS and those who did not (7 days, IQR:4,12 versus 10 days, IQR:6,20, p=0.2). The most common cause of death was respiratory failure both in those who died with ARDS and those who did not develop ARDS (p=0.4) (Figure E2).

Among the 76 patients who died in the hyperinflammatory group, 55 (72%) developed ARDS. The proportion of patients admitted with Full code status was higher in the patients who developed ARDS than those who did not (81% versus 71%, p=0.009) (Table E5). The SOFA score was higher in the patients who developed ARDS (13, IQR:11,15 versus 9, IQR:7-13, p=0.046), driven by a higher respiratory SOFA score. Time to death was not significantly different in patients who developed ARDS and those who did not (6 days, IQR:2,13 days versus 7 days, IQR:2,16 days, respectively, p=0.8). The main cause of death was circulatory shock in both those who died with ARDS and those who did not develop ARDS (p=0.13) (Figure E2).

# E-Tables

## Table E1: Definition of severe and irreversible organ system dysfunction derived from Stapleton et al. and Ketcham et al.

| System failure | Severe | Irreversible |
| --- | --- | --- |
| Circulatory | Sustained hypotension (mean arterial pressure <65 mmHg) requiring vasopressors | Mean arterial pressure <65 mmHg with unresponsiveness to vasopressors (refractory shock). Option was given to apply irreversible dysfunction if care was withdrawn due to poor prognosis related to shock. |
| Pulmonary | Inability to liberate from any ventilatory support outside of neurological context | Insupportable oxygenation or ventilation defined as PaO2<40 mmHg on FiO2=1 for >2 hours or respiratory acidosis with pH <7.1 on maximum ventilatory settings |
| Neurologic | Glasgow coma scale <8 in patient with identified causes of coma over 3 days after stopping sedation | Meet brain death criteria. Option was given to apply irreversible dysfunction if care was withdrawn due to poor prognosis related to neurologic organ system dysfunction |
| Hematologic | Microvascular bleeding with either fibrinogen <100 mg/dL, prothrombin time and partial thromboplastin time >1.5 times control, or platelets< 60,000/mm^3^ | Ongoing microvascular bleeding not surgically correctable with MAP<65 mmHg not reversible with blood products. Option was given to apply irreversible dysfunction if care was withdrawn due to poor prognosis related to hematologic organ system dysfunction. |
| Hemorrhage | MAP<65 mmHg for>2 hours or requiring vasopressors necessitating blood transfusion and excluding other causes of hypotension | Uncontrollable “surgical” bleeding from a non-micro-vascular source. Option was given to apply irreversible dysfunction if care was withdrawn related to hemorrhage |
| Hepatic | Bilirubin > 5 mg/dL and albumin<20g/dL and prothrombin time or partial thromboplastin time > 1.5 times control | Severe criteria plus hepatic encephalopathy and/or hepatorenal syndrome not responsive to treatment. Option was given to apply irreversible dysfunction if care was withdrawn due to poor prognosis related to hepatic organ system dysfunction |
| Gastrointestinal | Resectable ruptured or necrotic bowel, or pancreatitis causing shock | Inoperable ruptured or necrotic bowel or pancreatitis causing irreversible shock. Option was given to apply irreversible dysfunction if care was withdrawn due to poor prognosis related to gastrointestinal organ system dysfunction |
| Renal | Either creatinine > 5 mg/dL or requiring hemodialysis | Renal failure with acidosis, hyperkaliemia causing irreversible dysfunction if care was withdrawn due to poor prognosis related to renal organ system dysfunction |

## Table E2: Characteristics on ICU admission between EARLI and VALID cohort

|  | EARLI, N = 675 | VALID, N = 1062 | p-value |
| --- | --- | --- | --- |
| Age (years) | 66 (55, 78) | 58 (47, 67) | <0.001 |
| Male | 390 (58%) | 587 (55%) | 0.3 |
| Race |  |  | <0.001 |
| *African American* | 94 (14%) | 136 (13%) |  |
| *Asian* | 172 (26%) | 7 (1%) |  |
| *Caucasian (Non Hispanic)* | 293 (43%) | 907 (85%) |  |
| *Hispanic* | 32 (5%) | 10 (1%) |  |
| *Other* | 83 (12%) | 2 (0.2%) |  |
| Weight (kg) | 68 (56, 84) | 80 (66, 99) | <0.001 |
| SAPS II | 47 (35, 64) | 51 (39, 65) | 0.003 |
| Modified APACHE II | 23 (18, 30) | 25 (19, 30) | 0.12 |
| Diabetes | 158(23%) | 348 (33%) | <0.001 |
| Congestive heart failure | 155 (23%) | 140 (13%) | <0.001 |
| Coronary artery disease | 99 (15%) | 134 (13%) | 0.2 |
| Stroke | 54 (8%) | 96 (9%) | 0.5 |
| Chronic liver disease | 70 (10%) | 116 (11%) | 0.7 |
| Chronic kidney disease | 106 (16%) | 229 (22%) | 0.003 |
| Chronic dialysis | 49 (7%) | 59 (6%) | 0.2 |
| Vasopressors | 390 (58%) | 494 (47%) | <0.001 |
| Fluids administration received in Emergency department (L) | 2.0 (1.0,3.1) | 4.1 (2.2,6.6) | <0.001 |
| Albumin (g/L) | 2.3 (2.0,2.8) | 2.6 (2.3,3.0) | <0.001 |
| Hematocrit (%) | 29 (25, 34) | 29 (25, 34) | 0.7 |
| Creatinine (mg/l) | 1.37 (0.91, 2.39) | 1.59 (0.98, 2.89) | <0.001 |
| Bicarbonate (mmol/l) | 20.0 (17.0, 24.0) | 22.0 (19.0, 25.0) | <0.001 |
| Protein C (%) | 71 (38, 113) | 53 (35, 78) | <0.001 |
| IL-6 (pg/ml) | 101 (23, 729) | 57 (18, 241) | <0.001 |
| IL-8 (pg/ml) | 18 (8, 83) | 21 (9, 71) | 0.2 |
| Invasive Mechanical ventilation on enrollment | 307 (45%) | 643 (61%) | <0.001 |
| Pulmonary sepsis | 379 (56%) | 510 (48%) | <0.001 |
| Phenotype of sepsis |  |  | 0.015 |
| Hypoinflammatory | 431 (64%) | 737 (69%) |  |
| Hyperinflammatory | 244 (36%) | 325 (31%) |  |
| ARDS* | 320 (47%) | 392 (37%) | <0.001 |
| PaO2/FiO2 (mmHg) | 164 (92, 242) | 160 (97, 255) | 0.4 |
| SpO2/FiO2 ‡ | 262 (189, 334) | 188 (118, 257) | <0.001 |
| ARDS severity† |  |  | <0.001 |
| *No ARDS* | 355 (53%) | 670 (63%) |  |
| *Mild* | 83 (12%) | 80 (8%) |  |
| *Moderate* | 114 (17%) | 167 (16%) |  |
| *Severe* | 123 (18%) | 145 (14%) |  |
| ICU stay of length (days) $ | 3 (2, 6) | 5 (3, 10) | <0.001 |
| Ventilation length (days) $ | 0 (0, 3) | 2 (0, 5) | <0.001 |
| In hospital mortality to 60 days | 179 (27%) | 268 (25%) | 0.6 |

* Proportion of patients who developed ARDS within the five days of assessment

† At the time of ARDS onset

‡ Collected only when Pao2/FiO2 was absent

$ For survivors only

## Table E3: Estimation of population attributable fraction of death from ARDS in each subphenotype of sepsis

|  | Hypoinflammatory subphenotype | | Hyperinflammatory subphenotype | |
| --- | --- | --- | --- | --- |
|  | **Population AF_ARDS_** | **CI95%** | **Population AF_ARDS_** | **CI95%** |
| Primary analysis using strata method on modified APACHE II score | 19% | 10,28 | 14% | 6,20 |
|  |  |  |  |  |
| Sensitivity analysis using strata method on modified APACHE II score |  |  |  |  |
|  |  |  |  |  |
| *Excluding probability in LCA<80%* | 21% | 11,31 | 14% | 6,22 |
| *Excluding patients>80 years* | 19% | 8,29 | 12% | 4,20 |
| *EARLI cohort only* | 19% | 3,33 | 16% | 2,28 |
| *VALID cohort only* | 18% | 6,29 | 15% | 5,24 |
| Sensitivity analysis using propensity score matching |  |  |  |  |
| *Adjusted for APACHE II, shock, cirrhosis, diabetes, pulmonary infection* | 20% | 7,32 | 11% | -1,21 |

## Table E4: Estimation of attributable fraction of death from ARDS in each subphenotype of sepsis

|  | Hypoinflammatory subphenotype | | Hyperinflammatory subphenotype | |
| --- | --- | --- | --- | --- |
|  | **AF_ARDS_** | **CI95%** | **AF_ARDS_** | **CI95%** |
| Primary analysis | 36% | 24,45 | 23% | 14,31 |
|  |  |  |  |  |
| Sensitivity analysis using strata method on modified APACHE II score |  |  |  |  |
| *Excluding probability in LCA<80%* | 38% | 26,48 | 24% | 15,32 |
| *Excluding patients>80 years* | 36% | 22,46 | 21% | 11,30 |
| *EARLI cohort only* | 33% | 11,46 | 23% | 9,33 |
| *VALID cohort only* | 36% | 19,47 | 29% | 17,39 |
| Sensitivity analysis using propensity score matching |  |  |  |  |
| *Adjusted for APACHE II, shock, cirrhosis, diabetes, pulmonary infection* | 36% | 16,51 | 19% | -1,36 |

## Table E5: Details of the SOFA score without neurologic component before the day of death or at the time of withdrawal of life support, stratified by phenotype

|  | Overall sepsis (N=130) | | | ARDS subgroup (N=85) | | |
| --- | --- | --- | --- | --- | --- | --- |
|  | Hypoinflammatory  N = 54 | Hyperinflammatory  N = 76 | p-value | Hypoinflammatory  N = 30 | Hyperinflammatory  N = 55 | p-value |
| Cardiovascular component of SOFA | 0 (0,0) | 4 (0,4) | <0.001 | 0 (0,0) | 4 (3,4) | <0.001 |
| Respiratory component of SOFA | 2 (1,3) | 2 (2,3) | 0.3 | 2 (2,3) | 3 (2,3) | 0.8 |
| Coagulation component of SOFA | 0 (0,1) | 2(1,3) | <0.001 | 0 (0,2) | 2 (1,3) | <0.001 |
| Liver component of SOFA | 0 (0,1) | 1 (0,2) | <0.001 | 0 (0,1) | 1 (0,2) | 0.003 |
| Renal component of SOFA | 2 (0,4) | 4 (2,4) | <0.001 | 3(0,4) | 4 (2,4) | 0.019 |

## Table E6: Characteristics of patients before the day of death or at the time of the withdrawal of life support, stratified by subphenotype and presence or not of ARDS

|  | Hypoinflammatory sepsis (N=54) | | | Hyperinflammatory sepsis (N=76) | | |
| --- | --- | --- | --- | --- | --- | --- |
|  | No ARDS  N = 24 | ARDS  N = 30 | p-value | No ARDS  N = 21 | ARDS  N = 55 | p-value |
| Full code on admission | 15 (62%) | 17 (57%) | >0.9 | 15 (71%) | 44 (81%) | 0.009 |
| Withdrawal of life support decision | 16 (73%) | 22 (79%) | 0.6 | 11 (58%) | 39 (75%) | 0.2 |
| Modified SOFA score^1^ | 4 (2, 8) | 6 (4, 8) | 0.2 | 9 (7, 13) | 13 (11, 15) | 0.046 |
| Cardiovascular SOFA | 0 (0,1) | 0 (0,0) | 0.3 | 4 (0,4) | 4 (3,4) | 0.2 |
| Respiratory SOFA | 2 (0,2) | 2 (2,3) | 0.011 | 1 (1,3) | 3 (2,3) | 0.025 |
| Coagulation SOFA | 0 (0,1) | 0 (0,2) | >0.9 | 2 (1,3) | 2 (1,3) | >0.9 |
| Liver SOFA | 0 (0,1) | 0 (0,1) | >0.9 | 2 (0,2) | 1 (0,2) | >0.9 |
| Renal SOFA | 1 (0,4) | 3 (0,4) | 0.5 | 4 (2,4) | 4 (2,4) | 0.6 |
| Multiorgan failure | 16 (67%) | 18 (75%) | 0.5 | 17 (81%) | 47 (89%) | 0.5 |
| Time from ICU admission to death (days) | 10 (6, 20) | 7 (4, 12) | 0.2 | 7 (2, 16) | 6 (2, 13) | 0.8 |
| Main organ failure involved in the death |  |  | 0.4 |  |  | 0.2 |
| *Circulatory failure* | 9 (38%) | 6 (20%) |  | 11 (52%) | 37 (67%) |  |
| *Respiratory failure* | 12 (50%) | 20 (67%) |  | 2 (9.5%) | 9 (16%) |  |
| *CNS* | 2 (8.3%) | 3 (10%) |  | 1 (4.8%) | 3 (5.5%) |  |
| *GI* | 1 (4.2%) | 0 (0%) |  | 3 (14%) | 2 (3.6%) |  |
| *Hemorrhage* | 0 (0%) | 0 (0%) |  | 1 (4.8%) | 0 (0%) |  |
| *Hepatic* | 0 (0%) | 0 (0%) |  | 3 (14%) | 2 (3.6%) |  |
| *Renal* | 0 (0%) | 1 (3.3%) |  | 0 (0%) | 1 (1.8%) |  |
| *Hematologic* | 0 (0%) | 0 (0%) |  | 0 (0%) | 1 (2%) |  |
| *^1^* Without neurological component | | | | | | |

# E-Figures

**Figure E1:** Algorithm from *Ketcham et al.* (PMID 32620175) to determinate the primary cause of death

**Figure E2:** Directed Acyclic graph used for propensity score matching.

**
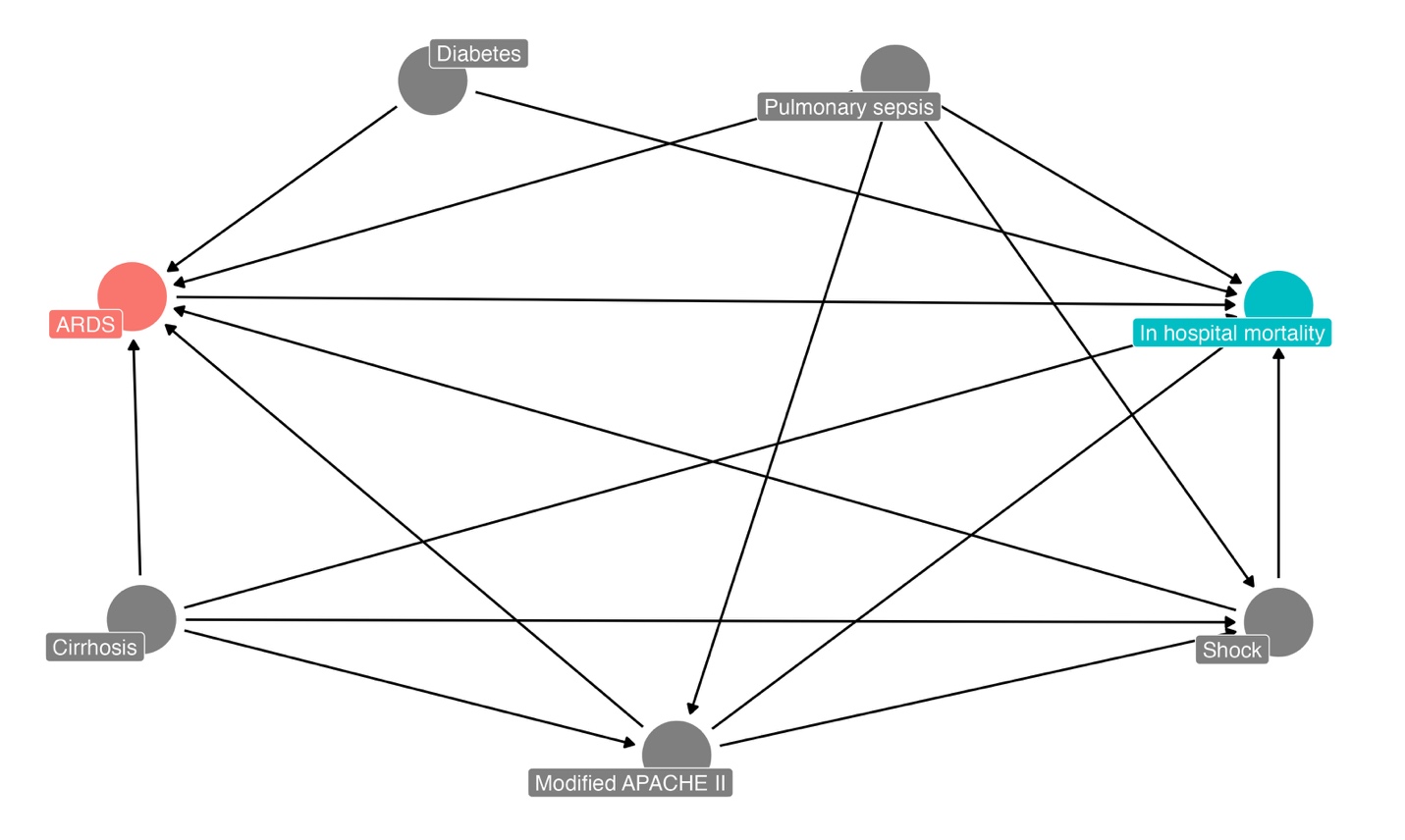
**

**Figure E3:** A.Barplot showing the day of diagnosis of ARDS from ICU admission (Day 1) in each subphenotype of sepsis. Proportion in % of total ARDS in each phenotype is provided. B. Barplot showing the proportion of patient who died in each phenotype and stratified by the timing of death. Proportion in % of total death in each phenotype is provided.

*Abbreviations: ARDS; Acute Respiratory Distress Syndrome*

**
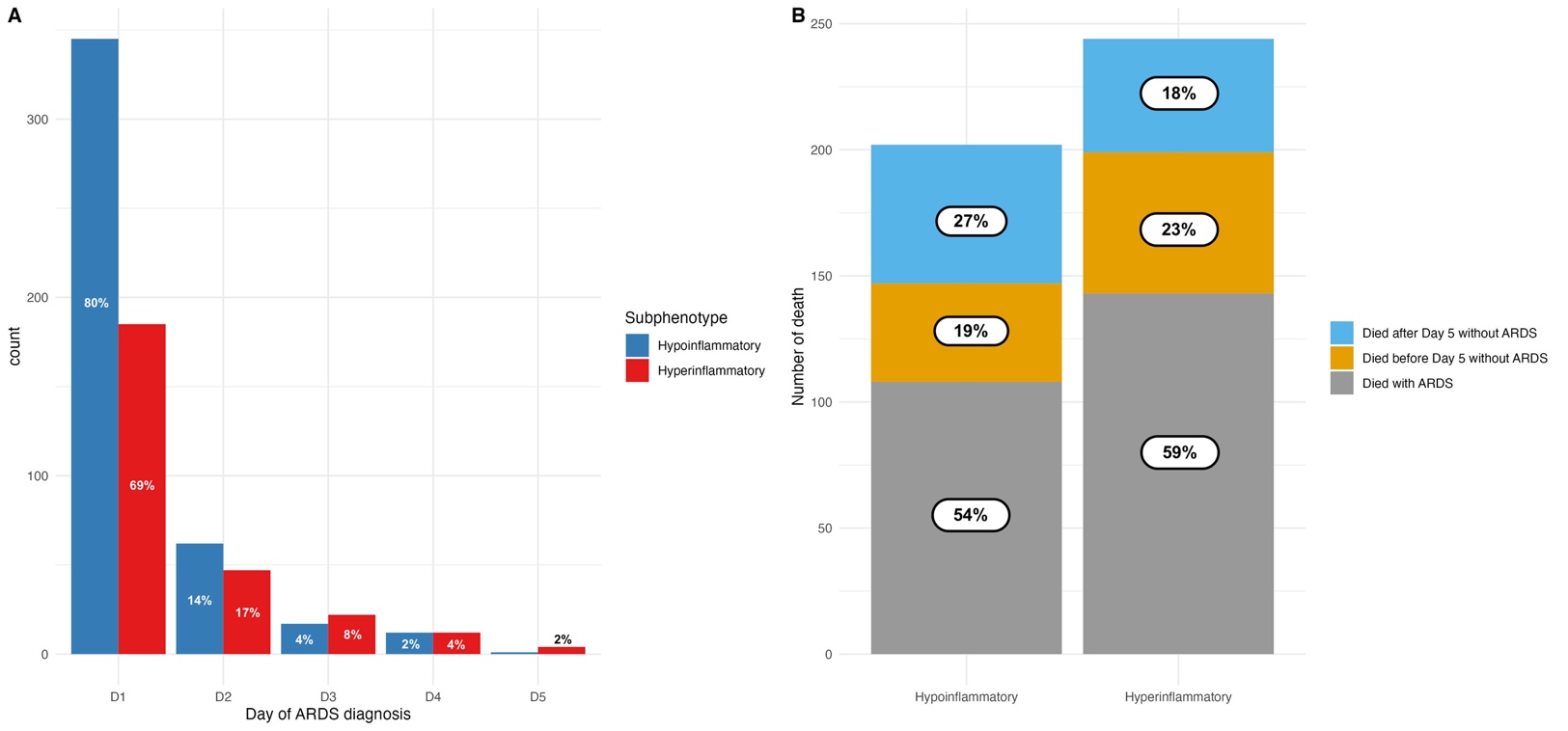
**

**Figure E4:** Overview of the study

**Figure E5:** Alluvial plot showing the relation between severe comorbidities, the origin of sepsis the phenotype of sepsis, the presence or not of ARDS and the cause of death, stratified by the subphenotype of sepsis. Hypoinflammatory sepsis ***(in blue)*** was mainly due to pulmonary origin whereas hyperinflammatory sepsis ***(in red)*** was mainly to extra-pulmonary origin.

*Abbreviations: ARDS, Acute respiratory distress syndrome*


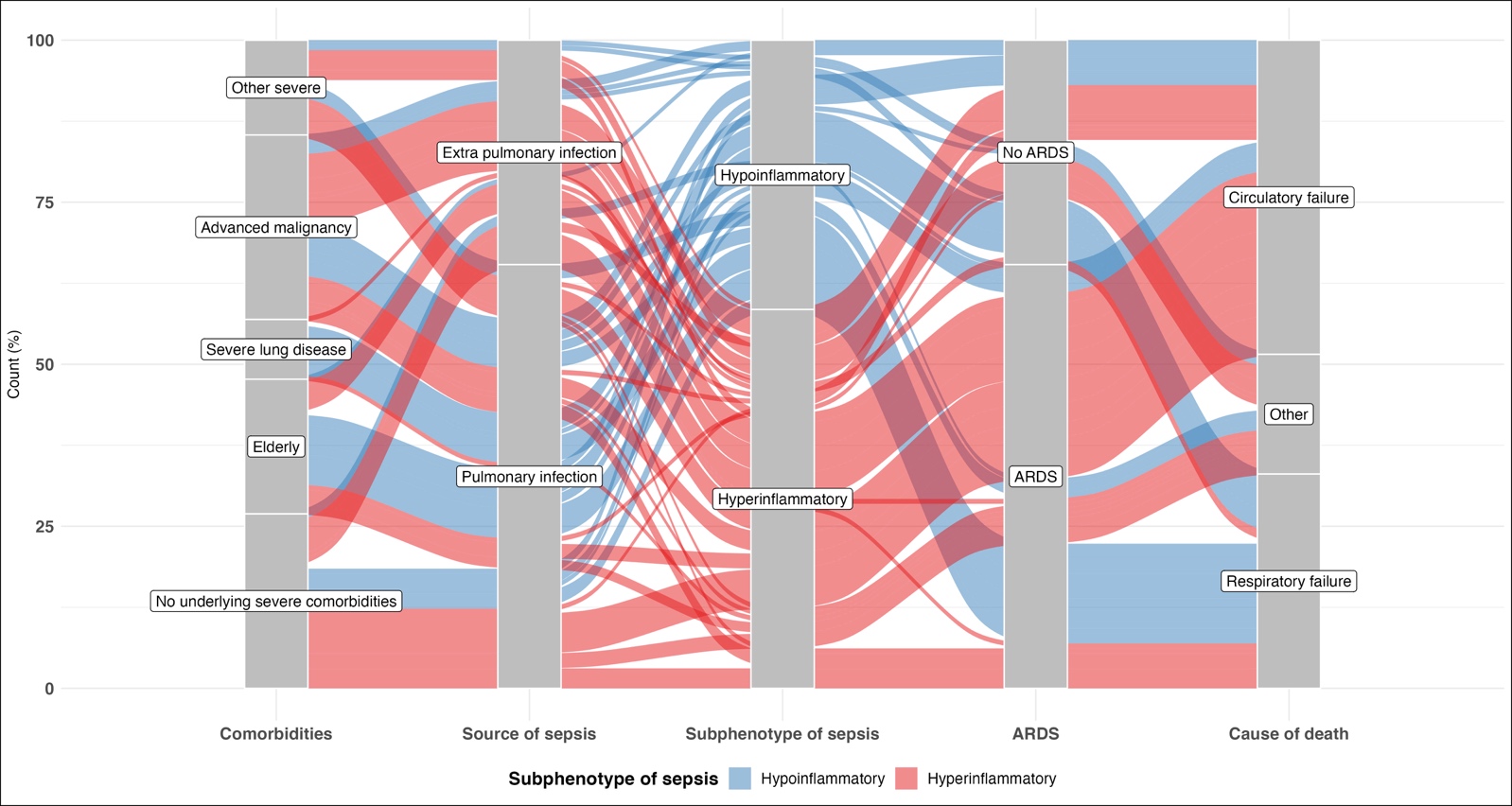


**Figure E6:** Upset plot showing the number of patients with one or multiple irreversible (left part) or severe organ dysfunction (right part) collected at the time of death or the withdrawal of life support in hypoinflammatory sepsis using the standardized case ascertainment template. For example, 18 patients had only severe respiratory organ dysfunction, 16 had both severe respiratory and circulatory dysfunction criteria and no one had multiple irreversible organ dysfunction. Respiratory failure was never driven by irreversible hypoxemia but mainly by failure to wean from ventilatory or oxygenation support. The most frequent severe organ dysfunction associated with respiratory dysfunction was circulatory failure and multiorgan failure was rare (right part).

*Abbreviations: None*

*
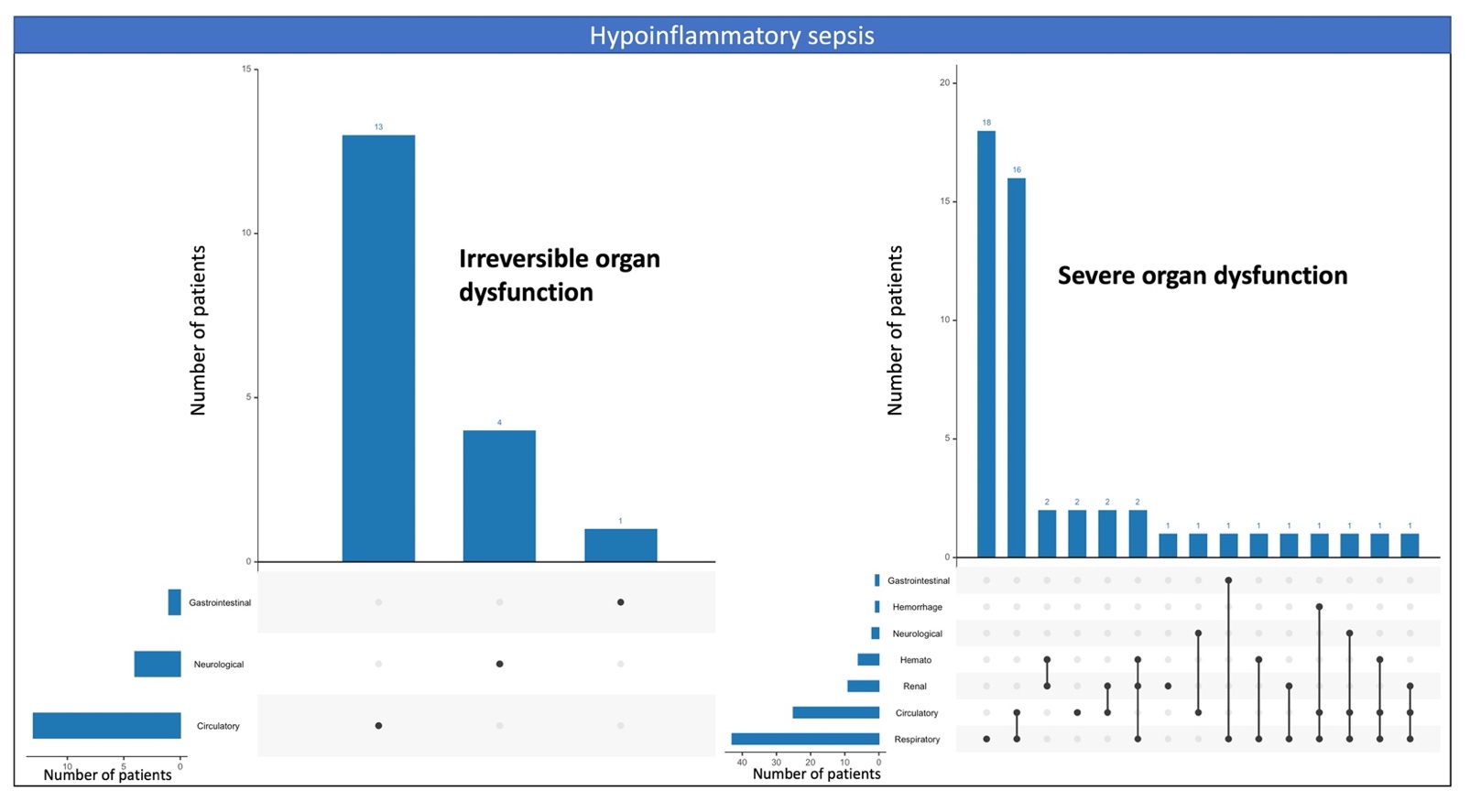
*

**Figure E7:**

Upset plot showing the number of patients with one or multiple irreversible (left part) or severe organ dysfunction (right part) collected at the time of death or the withdrawal of life support in hyperinflammatory sepsis using the standardized case ascertainment template. For example, 35 patients had only irreversible circulatory dysfunction, and 2 had both irreversible respiratory and circulatory dysfunction. Likewise, 5 patients had at the same time severe renal, circulatory and respiratory dysfunction. Irreversible circulatory dysfunction was highly represented. Only 2 patients had an irreversible respiratory dysfunction, which was always associated with irreversible circulatory dysfunction (left part). Almost all the patients developed multiorgan failure (right part).

*Abbreviations: None*


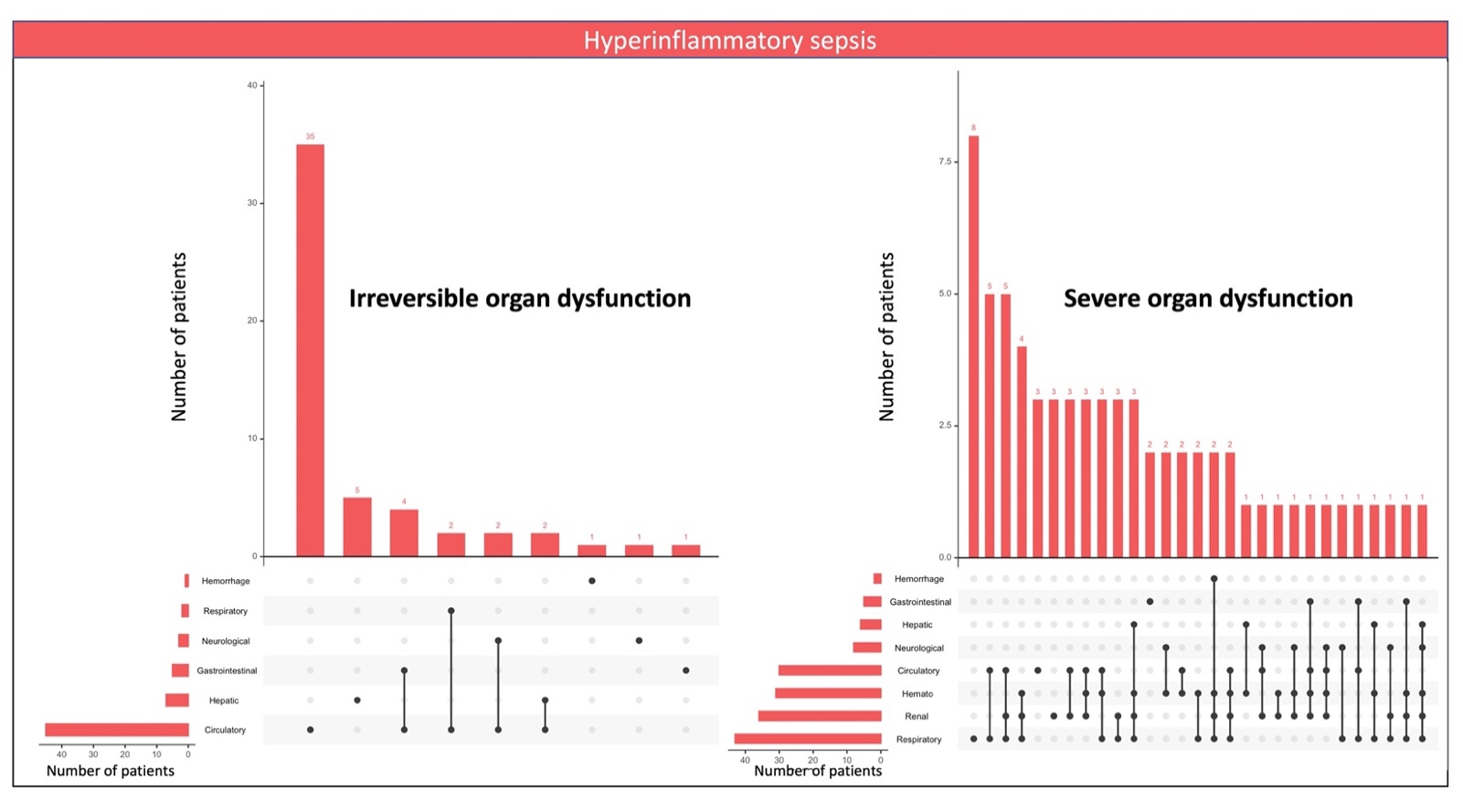


## Figure E**8:**

Results for the matching using propensity score in the hypoinflammatory sepsis. **A.**Love plot showing the standardized mean difference before and after matching. **B to G** Distributional balance represented by histograms or cumulative density function for each covariates and the propensity score before and after matching.

*Abbreviations: None*


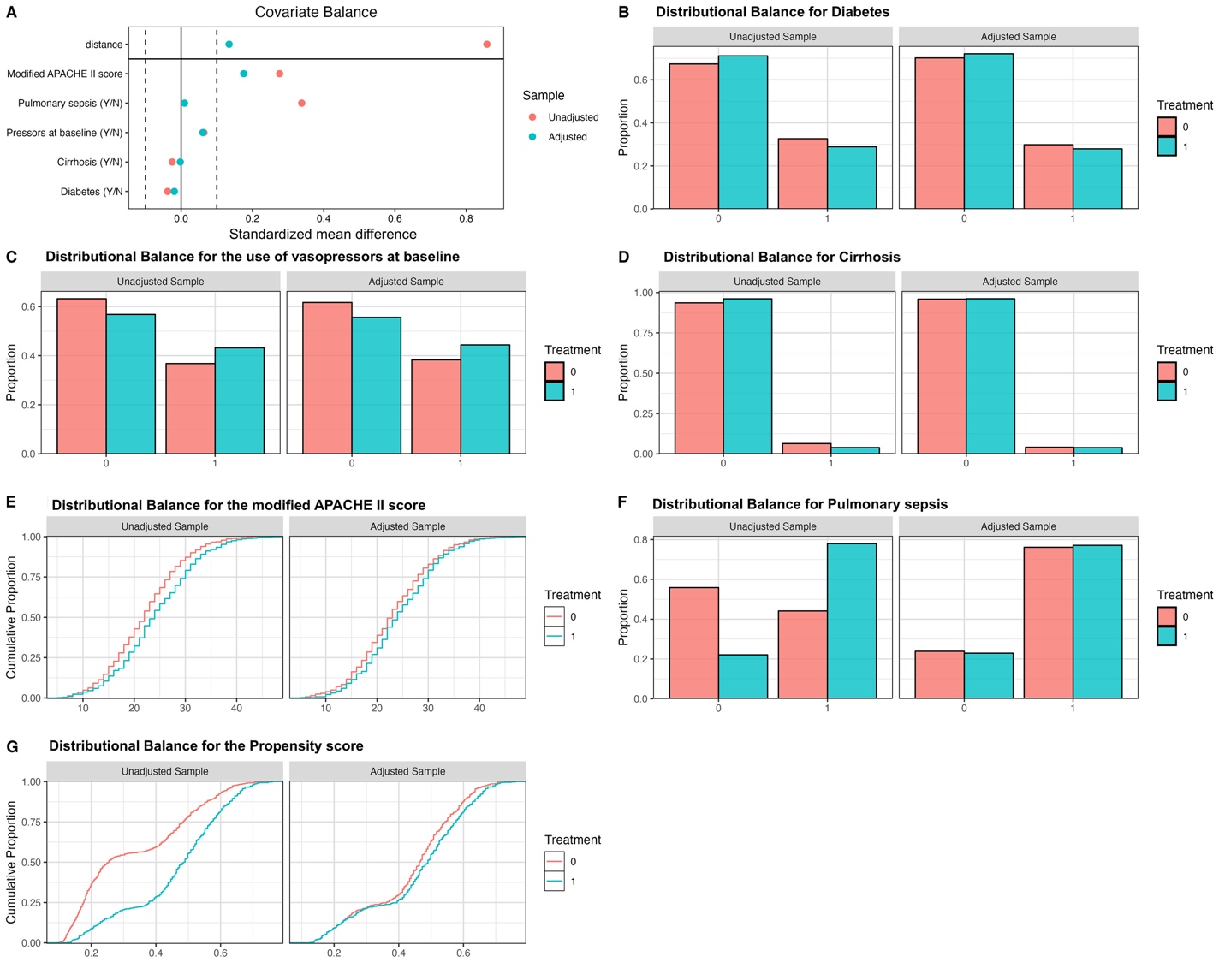


## **Figure E9:**

Results for the matching using propensity score in the hyperinflammatory sepsis. **A.**Love plot showing the standardized mean difference before and after matching. **B to G** Distributional balance represented by histograms or cumulative density function for each covariates and the propensity score before and after matching.

*Abbreviations: None*


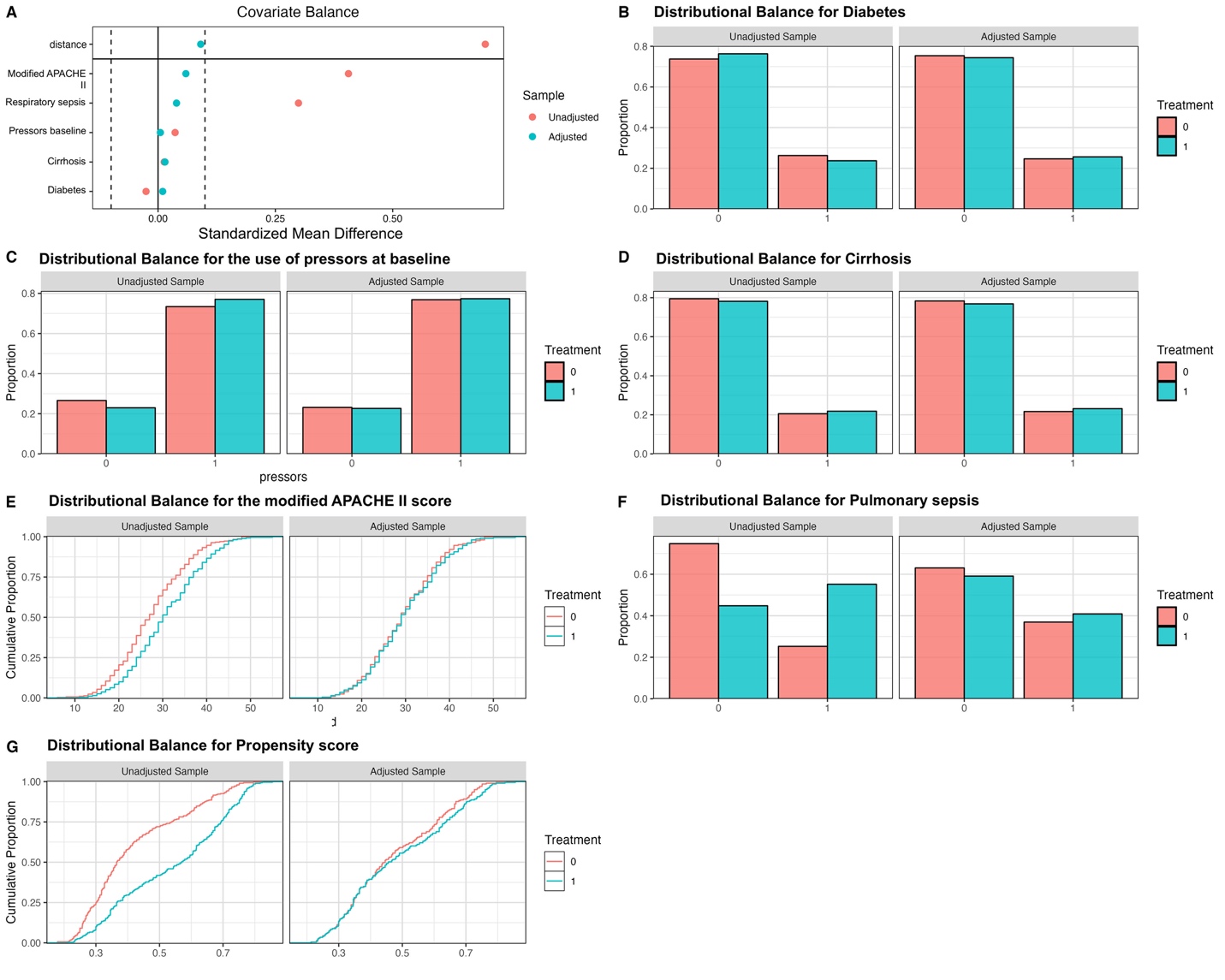


# E-references

1. Sinha P, Kerchberger VE, Willmore A, et al Identifying molecular phenotypes in sepsis: an analysis of two prospective observational cohorts and secondary analysis of two randomised controlled trials. The Lancet Respiratory Medicine. https://doi.org/10.1016/S2213-2600(23)00237-0

2. Sinha P, Delucchi KL, Chen Y, et al (2022) Latent class analysis-derived subphenotypes are generalisable to observational cohorts of acute respiratory distress syndrome: a prospective study. Thorax 77:13–21. https://doi.org/10.1136/thoraxjnl-2021-217158

3. Shah CV, Lanken PN, Localio AR, et al (2010) An alternative method of acute lung injury classification for use in observational studies. Chest 138:1054–1061. https://doi.org/10.1378/chest.09-2697

4. Stapleton RD, Wang BM, Hudson LD, et al (2005) Causes and Timing of Death in Patients With ARDS. Chest 128:525–532. https://doi.org/10.1378/chest.128.2.525

5. Ketcham SW, Sedhai YR, Miller HC, et al (2020) Causes and characteristics of death in patients with acute hypoxemic respiratory failure and acute respiratory distress syndrome: a retrospective cohort study. Crit Care 24:391. https://doi.org/10.1186/s13054-020-03108-w

6. Browner WS, Newman TB (1989) Sample size and power based on the population attributable fraction. Am J Public Health 79:1289–1294. https://doi.org/10.2105/AJPH.79.9.1289

7. Massart N, Wattecamps G, Moriconi M, Fillatre P (2021) Attributable mortality of ICU acquired bloodstream infections: a propensity-score matched analysis. Eur J Clin Microbiol Infect Dis 40:1673–1680. https://doi.org/10.1007/s10096-021-04215-4

# STROBE Statement

|  | Item No | Recommendation | Page No |
| --- | --- | --- | --- |
| **Title and abstract** | 1 | (*a*) Indicate the study’s design with a commonly used term in the title or the abstract | 1 |
|  |  | (*b*) Provide in the abstract an informative and balanced summary of what was done and what was found | 3 |
| Introduction | | | |
| Background/rationale | 2 | Explain the scientific background and rationale for the investigation being reported | 5-6 |
| Objectives | 3 | State specific objectives, including any prespecified hypotheses | 6 |
| Methods | | | |
| Study design | 4 | Present key elements of study design early in the paper | 6 |
| Setting | 5 | Describe the setting, locations, and relevant dates, including periods of recruitment, exposure, follow-up, and data collection | 6-7 |
| Participants | 6 | (*a*) Give the eligibility criteria, and the sources and methods of selection of participants. Describe methods of follow-up | 7 and E-methods |
|  |  | (*b*) For matched studies, give matching criteria and number of exposed and unexposed | E-methods |
| Variables | 7 | Clearly define all outcomes, exposures, predictors, potential confounders, and effect modifiers. Give diagnostic criteria, if applicable | 7 |
| Data sources/ measurement | 8* | For each variable of interest, give sources of data and details of methods of assessment (measurement). Describe comparability of assessment methods if there is more than one group | 7 and E-methods |
| Bias | 9 | Describe any efforts to address potential sources of bias | NA |
| Study size | 10 | Explain how the study size was arrived at | E-methods |
| Quantitative variables | 11 | Explain how quantitative variables were handled in the analyses. If applicable, describe which groupings were chosen and why | 8 |
| Statistical methods | 12 | (*a*) Describe all statistical methods, including those used to control for confounding | 8 and E-methods |
|  |  | (*b*) Describe any methods used to examine subgroups and interactions | 8 and E-methods |
|  |  | (*c*) Explain how missing data were addressed | 8 and E-methods |
|  |  | (*d*) If applicable, explain how loss to follow-up was addressed | NA |
|  |  | (*e*) Describe any sensitivity analyses | 8 and E-methods |
| Results | | |  |
| Participants | 13* | (a) Report numbers of individuals at each stage of study—eg numbers potentially eligible, examined for eligibility, confirmed eligible, included in the study, completing follow-up, and analysed | 9 and figure 1 |
|  |  | (b) Give reasons for non-participation at each stage | 9 and figure 1 |
|  |  | (c) Consider use of a flow diagram | Figure 1 |
| Descriptive data | 14* | (a) Give characteristics of study participants (eg demographic, clinical, social) and information on exposures and potential confounders | 9, tables, figures and supplement |
|  |  | (b) Indicate number of participants with missing data for each variable of interest | NA |
|  |  | (c) Summarise follow-up time (eg, average and total amount) | 9 |
| Outcome data | 15* | Report numbers of outcome events or summary measures over time | 9-11 and tables, figures and supplement |

*Give information separately for cases and controls in case-control studies and, if applicable, for exposed and unexposed groups in cohort and cross-sectional studies.

**Note:** An Explanation and Elaboration article discusses each checklist item and gives methodological background and published examples of transparent reporting. The STROBE checklist is best used in conjunction with this article (freely available on the Web sites of PLoS Medicine at http://www.plosmedicine.org/, Annals of Internal Medicine at http://www.annals.org/, and Epidemiology at http://www.epidem.com/). Information on the STROBE Initiative is available at [www.strobe-statement.org](http://www.strobe-statement.org).
